# The optimal dose of strength and conditioning training for enhancing physical performance in football players: a systematic review and network meta-analysis of randomized clinical trials

**DOI:** 10.3389/fphys.2026.1791257

**Published:** 2026-07-06

**Authors:** Shuyuan Du, Xinyi Li, Sheng-qiang Wang

**Affiliations:** 1 Department of Strength and Conditioning Training, Beijing Sport University, Beijing, China; 2 Department of China Medical System Holdings Limited, Shenzhen, Guangdong, China; 3 Department of Rehabilitation Medicine, Tongji Hospital, Tongji Medical College, Huazhong University of Science and Technology, Wuhan, China

**Keywords:** dose-response relationship, football players, meta-analysis, physical performance, strength and conditioning

## Abstract

**Objective:**

This study aims to evaluate the effects of four types of physical training interventions on the athletic performance of soccer players and to determine the optimal training dosage for each modality.

**Design:**

A systematic search of PubMed, EMBASE, Web of Science, the Cochrane Library, and OVID databases was conducted to identify randomized controlled trials (RCTs) examining the impact of these four exercise-based interventions on soccer performance. The methodological quality of the included studies was assessed using the Cochrane Risk of Bias tool, and all extracted data were analyzed using appropriate statistical software.

**Results:**

The RCTs included in this review were published from the earliest available records through September 2025. A total of fifty-three RCTs with an aggregated sample of 1,580 participants met the eligibility criteria. The network meta-analysis demonstrated that strength training produced the most substantial improvements in several performance indicators, including Counter Movement Jump (CMJ) (SUCRA: 94%), Squat Jump (SJ) (SUCRA: 87.5%), one-repetition maximum squat (1-RM squat) (SUCRA: 99.9%), five-meter sprint (5m-sprint) (SUCRA: 99.6%), ten-meter sprint (10m-sprint) (SUCRA: 87.4%), thirty-meter sprint (30m-sprint) (SUCRA: 88.8%), and agility measured by the T-test (SUCRA: 69.4%). Additionally, endurance training showed the greatest effect on maximal oxygen uptake (VO_2_max) (SUCRA: 84.7%).

**Conclusion:**

Based on the ranking plots generated by the network meta-analysis, strength training demonstrates superior overall benefits compared with other exercise interventions for enhancing the performance of football players.

## 1 Introduction

In the context of global competitive sports, soccer has emerged as one of the most intensely followed disciplines due to its high-intensity and highly confrontational nature (Hamm et al., 2018; Elbanna et al., 2025). A standard match lasts 90 minutes, with an additional 30-minute overtime period in knockout stages, during which players must continuously perform complex movements across a playing area of approximately 7,140 m² (Ventura et al., 2024). These actions include repeated short sprints of 10–30 meters—amounting to 30–60 sprints per match in elite competitions—frequent rapid decelerations and directional changes exceeding 50 occurrences per hour, as well as jumping duels, physical contests, and long-distance passes. Statistical reports indicate that professional players typically cover 7–10 kilometers of high-intensity running per match, with high-speed running (≥25 km/h) comprising 15–20% of the total distance (Di Salvo et al., 2007; Rampinini et al., 2007; Harper et al., 2019). This demanding activity profile imposes exceptional requirements on multiple components of physical fitness, including aerobic endurance (to sustain prolonged activity), anaerobic capacity (to accommodate repeated high-intensity sprints), muscular strength (to support jumping and physical contact), and agility (to enable rapid changes in direction) (Sarmento et al., 2024; Unji et al., 2024). As a result, physical conditioning has become a decisive factor influencing tactical execution, performance during critical moments, and ultimately, match outcomes. Consequently, the development of scientifically grounded training interventions aimed at precisely enhancing sport-specific physical qualities has become a central focus in soccer performance research.

The field of physical conditioning for soccer players currently faces three major challenges: fragmentation of training protocols, variability in training outcomes, and increasing complexity in performance evaluation (Morgans et al., 2014). Existing training approaches encompass multiple modalities, such as high-intensity interval training (HIIT), moderate-intensity continuous training (MICT), and power interval training (PIT), yet they differ markedly in key dosage parameters (Stankovic et al., 2023). For example, some HIIT protocols adopt a “30-second all-out sprint followed by 1-minute rest” pattern performed three times per week, whereas others employ “1 minute of high-intensity running followed by 2 minutes of recovery” performed twice weekly (Buchheit and Laursen, 2013). Such discrepancies in training parameters directly contribute to divergent outcomes. Several RCTs have demonstrated that HIIT can increase maximal oxygen uptake (VO_2_max) by 8%–12% and enhance anaerobic threshold velocity by 5%–8%, significantly improving players’ ability to sustain high-intensity running during the latter stages of competition (Faude et al., 2013; Bouali, 2025). Conversely, other studies have reported that when HIIT frequency exceeds four sessions per week or when individual sessions last more than 40 minutes, the incidence of muscle microdamage can increase by 30%, and the recovery period may extend by an additional 24–48 hours, ultimately compromising subsequent training quality (Hostrup and Bangsbo, 2023; Saif et al., 2025). Athlete heterogeneity further complicates program selection: youth athletes (12–18 years), whose musculoskeletal systems are still developing, exhibit only 60%–70% of the tolerance displayed by adults when exposed to training intensities exceeding 85% of maximal heart rate (Malina et al., 2015). Professional athletes, whose primary objective is performance enhancement, may train 4–6 hours daily, whereas recreational athletes typically train less than 5 hours per week due to time and energy constraints, resulting in fundamentally different requirements for training intensity and frequency (Nédélec et al., 2012). Moreover, inconsistencies in evaluation metrics—some studies prioritizing total running distance, others emphasizing sprint speed improvements or lactate clearance—have led to a fragmented assessment framework, making it increasingly difficult to conduct meaningful comparisons across training interventions (Edgecomb and Norton, 2006; Faude et al., 2009).

The limitations of traditional research methodologies have long constrained efforts to determine the optimal training dosage for soccer players, resulting in a landscape characterized by extensive localized investigations but limited systematic integration. Although RCTs provide high-quality evidence, individual studies typically compare only two or three training modalities (e.g., “HIIT vs MICT”) and often involve small sample sizes of 20–50 participants, thereby failing to adequately represent athletes of varying ages and competitive levels or to comprehensively examine multidimensional dosage interactions across intensity, frequency, and duration (Guyatt et al., 2008). Conventional meta-analyses, while capable of synthesizing findings from multiple RCTs to enhance statistical power, remain restricted by their analytical framework, which only permits pairwise comparisons (Higgins et al., 2013). As such, they are unable to accommodate more complex scenarios involving simultaneous comparisons of multiple interventions. For instance, evaluating the relative effectiveness of HIIT, MICT, and PIT would require three separate meta-analyses—”HIIT vs MICT,” “HIIT vs PIT,” and “MICT vs PIT”—and still would not leverage indirect evidence (e.g., inferring “HIIT vs PIT” from “HIIT vs MICT” and “MICT vs PIT”), leading to inefficiencies and suboptimal utilization of available data (Dias et al., 2010; Lumley, 2010). In contrast, network meta-analysis (NMA), an advanced statistical approach widely applied in evidence-based research, offers three distinct advantages: (1) the ability to incorporate multiple interventions simultaneously (including more than five training dosage configurations), thereby constructing a comprehensive network of intervention–outcome relationships; (2) the integration of both direct comparisons (e.g., studies directly comparing HIIT and MICT) and indirect comparisons (e.g., effects inferred through a common comparator), substantially improving the completeness of the evidence base; and (3) the use of Bayesian hierarchical modeling to quantify sampling variability (such as errors arising from differences in sample sizes), intervention heterogeneity (such as discrepancies in training parameters), and between-study inconsistency (such as variations in outcome measures), ultimately generating probability-based rankings of intervention effectiveness (e.g., “HIIT protocol A has a 92% probability of producing the greatest improvement in VO_2_max compared with other protocols”) (Antoniou et al., 2019; Belavý et al., 2024; Perren et al., 2025).

**Figure 1:**
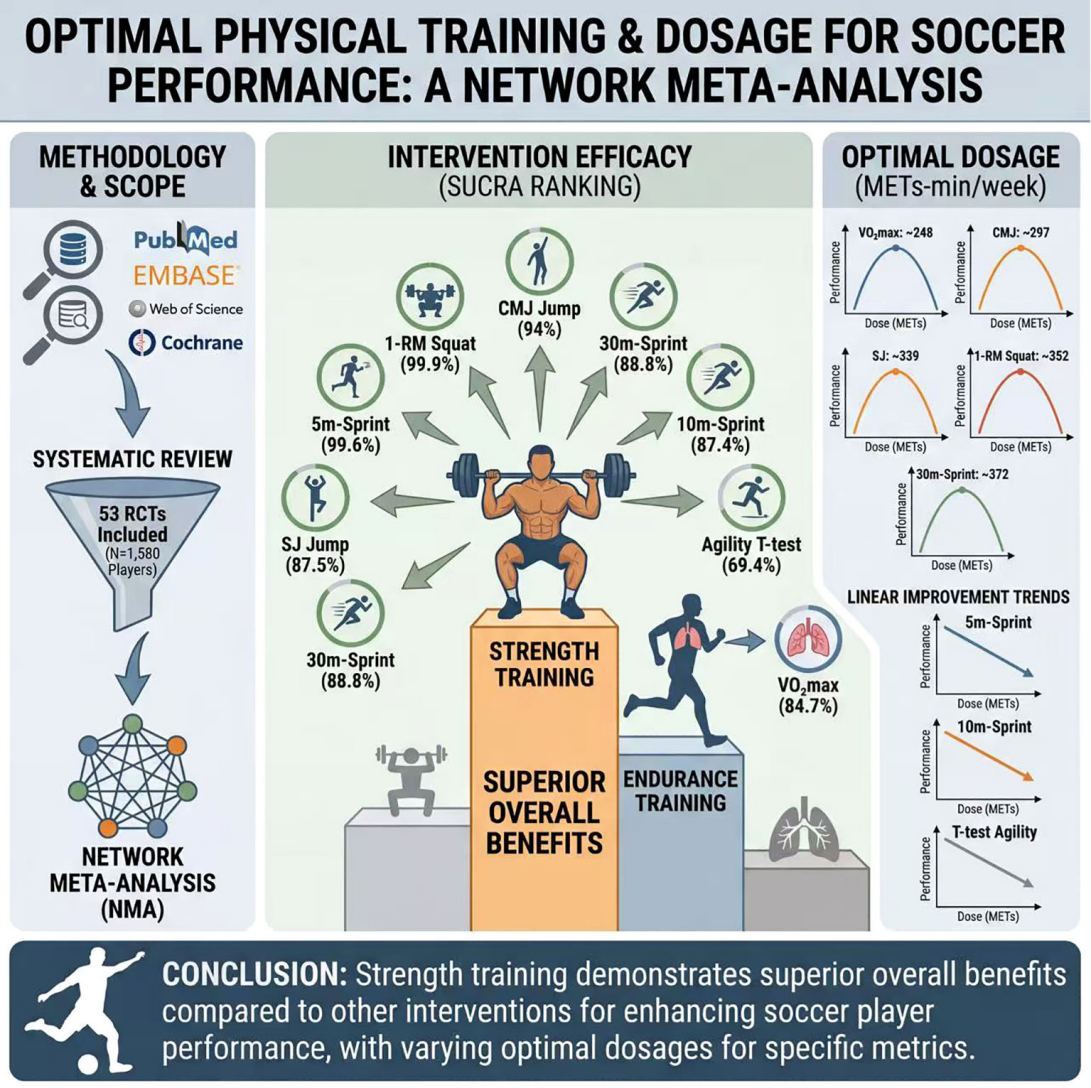
Research prospectus diagram.

In summary, the sport-specific physical demands of soccer, the fragmented nature and uncertain effectiveness of existing training protocols, and the methodological limitations of traditional research collectively underscore the necessity of conducting a systematic review and network meta-analysis. By systematically retrieving relevant studies worldwide and applying an NMA framework to integrate multidimensional evidence, the present study aims to precisely elucidate the relationships between various training dosages (intensity, frequency, and duration) and key performance outcomes in soccer players (such as endurance, speed, and explosive power). This approach not only addresses the existing gap regarding the optimal dosage of soccer-specific physical training and provides a robust theoretical foundation for exercise science, but also generates practical guidance for coaching staff in designing individualized training regimens—for example, recommending a “moderate-intensity, high-frequency, short-duration” protocol for youth athletes, or a “high-intensity, moderate-frequency, long-duration” protocol for professional players (**Figure 1**). Ultimately, the findings of this study are expected to enhance training efficiency, reduce injury risk, and optimize competitive performance, thereby offering substantial theoretical and practical value.

## 2 Materials and methods

### 2.1 Search strategy

The researchers searched five electronic databases—PubMed, EMBASE, Web of Science, the Cochrane Central Register of Controlled Trials, and OVID—from their inception to September 2025. In accordance with the PICOS framework recommended by the Cochrane Collaboration, the inclusion criteria were as follows: (P) Population: participants were soccer players of any competitive level; (I) Intervention: physical training primarily targeting strength, speed, endurance, or agility; (C) Comparator: SSG or conventional training; (O) Outcomes: at least one of the following performance indicators—1RM squat, Countermovement Jump, Squat Jump, 5 m sprint, 10 m sprint, 30 m sprint, T test, or VO_2_max test; and (S) Study type: RCTs. Specific details are provided in **Supplementary Table 1**.

### 2.2 Inclusion criteria

The inclusion criteria for eligible studies were as follows: (1) the experimental group employed various forms of strength and conditioning training as interventions for soccer players; (2) the control group received only routine training or small-sided games; (3) the study design was a clinical randomized controlled trial; and (4) the outcome measures included at least one of the following performance indicators: CMJ, Squat Jump (SJ), one-repetition maximum squat (1-RM squat), five-meter sprint (5m-sprint), ten-meter sprint (10m-sprint), thirty-meter sprint (30m-sprint), T-test for agility (T test), or maximal oxygen uptake (VO_2_max).

### 2.3 Exclusion criteria

The exclusion criteria were as follows: (1) studies involving participants who were not soccer players; (2) non-RCT designs; (3) intervention durations shorter than two weeks; (4) studies lacking accessible outcome data; (5) non-original research such as reviews or articles relying on previously published experimental results; and (6) conference abstracts.

### 2.4 Study selection

The literature screening process was conducted using the reference management software Zotero. Two researchers independently performed an initial review of the titles to eliminate duplicates as well as non–randomized controlled trial studies, review articles, conference papers, study protocols, and correspondence. Subsequently, both researchers examined the abstracts to identify eligible studies and exclude those not meeting the criteria. The remaining articles were then subjected to full-text assessment by both reviewers to determine final eligibility. Throughout the entire process, the researchers screened the literature independently and compared their selections; studies identified consistently by both reviewers were included, whereas discrepancies were resolved through discussion and, when necessary, adjudication by a third researcher.

### 2.5 Data collection

A standardized, pre-designed data extraction form consisting of seven items was used to collect relevant information from the included studies. The extracted variables comprised the following: (1) author, (2) year of publication, (3) country, (4) study period, (5) sample size, (6) mean age of participants, and (7) detailed descriptions of the exercise interventions.

For each study, we additionally extracted detailed descriptions of the interventions, including the frequency, intensity, type, and duration of each training session. Using the Compendium of Physical Activities, we calculated the energy expenditure dose for each exercise group, expressed as metabolic equivalent (MET) minutes per week. Furthermore, two authors independently evaluated each intervention using the Behavior Change Technique Taxonomy version 1 to identify the specific behavior change techniques explicitly incorporated into each exercise program.

### 2.6 Risk of bias of individual studies

Two researchers independently evaluated the risk of bias (ROB) using the Cochrane Handbook version 5.1.0 criteria for assessing ROB in RCTs. The assessment covered seven domains: (1) randomized sequence generation, (2) allocation concealment, (3) blinding of participants, (4) blinding of personnel, (5) completeness of outcome data, (6) selective reporting, and (7) other potential sources of bias. Based on the number of domains judged to have a high ROB, studies were classified into three categories: high risk (five or more domains), moderate risk (three or four domains), and low risk (two or fewer domains) (Higgins et al., 2011).

### 2.7 Data analysis

In studies where strength and conditioning served as the intervention, all outcome variables were continuous and reported as means with standard deviations (SD) (Li and Chen, 2021). Continuous outcomes were analyzed using either mean difference (MD), defined as the absolute difference in means between the experimental and control groups when measured on the same scale, or standardized mean difference (SMD), calculated as the mean difference between groups divided by the pooled standard deviation when outcomes were assessed using different measurement scales. Both effect sizes were presented with 95% confidence intervals (CI). Given the expected heterogeneity across studies, a random-effects model was selected for the analysis rather than a fixed-effects model (Jackson et al., 2011).

We used Stata software (version 15.1) to conduct the NMA and performed model aggregation and analysis through Markov chain Monte Carlo simulation within a Bayesian framework, following the PRISMA-NMA guidelines (Moher et al., 2015; Vats et al., 2019). The node-splitting method was applied to quantify and assess the consistency between direct and indirect comparisons, with calculations conducted according to the procedures implemented in Stata. A P-value greater than 0.05 was considered indicative of acceptable consistency between evidence sources (Salanti et al., 2011).

Stata software was used to generate and illustrate network diagrams representing the various exercise interventions. In these diagrams, each node corresponds to a specific training intervention or control condition, while the connecting lines indicate direct head-to-head comparisons between interventions. The size of each node and the thickness of each connecting line are proportional to the number of studies contributing to that respective intervention or comparison (Chaimani et al., 2013).

The hierarchy of interventions was summarized and reported using P scores, which serve as the frequentist analogue to surface under the cumulative ranking curve (SUCRA) values. A P score reflects the degree of certainty that one training intervention is superior to another, averaged across all competing interventions. P scores range from 0 to 1, with a value of 1 indicating the most effective intervention with no uncertainty and a value of 0 indicating the least effective intervention with no uncertainty. Although P scores or SUCRA values can be interpreted as representing the relative effectiveness or acceptability of exercise interventions, they should be approached cautiously unless the comparisons correspond to clinically meaningful differences (Marotta et al., 2020). To assess potential small-study effects that may introduce publication bias into the NMA, a network funnel plot was generated and visually examined for symmetry (Khera et al., 2016).

We analyzed the relationship between overall training dosage and athletic performance using the MBNMA dose–response package within the R statistical environment (version 4.2.2, www.r-project.org). Multiple model structures were explored, including the Emax function, restricted cubic splines (RCS), nonparametric models, and exponential models, each fitted under both random-effects and fixed-effects assumptions. To identify the best-fitting model, we compared several goodness-of-fit criteria, such as the deviance information criterion (DIC), standard deviations, model parameters, and residuals (Pedder, 2021). Based on these comparisons, the restricted cubic spline model with random effects demonstrated superior model performance; therefore, this model was selected for subsequent analyses (Rao, 2003).

## 3 Results

### 3.1 Study and identification and selection

**Figure 2:**
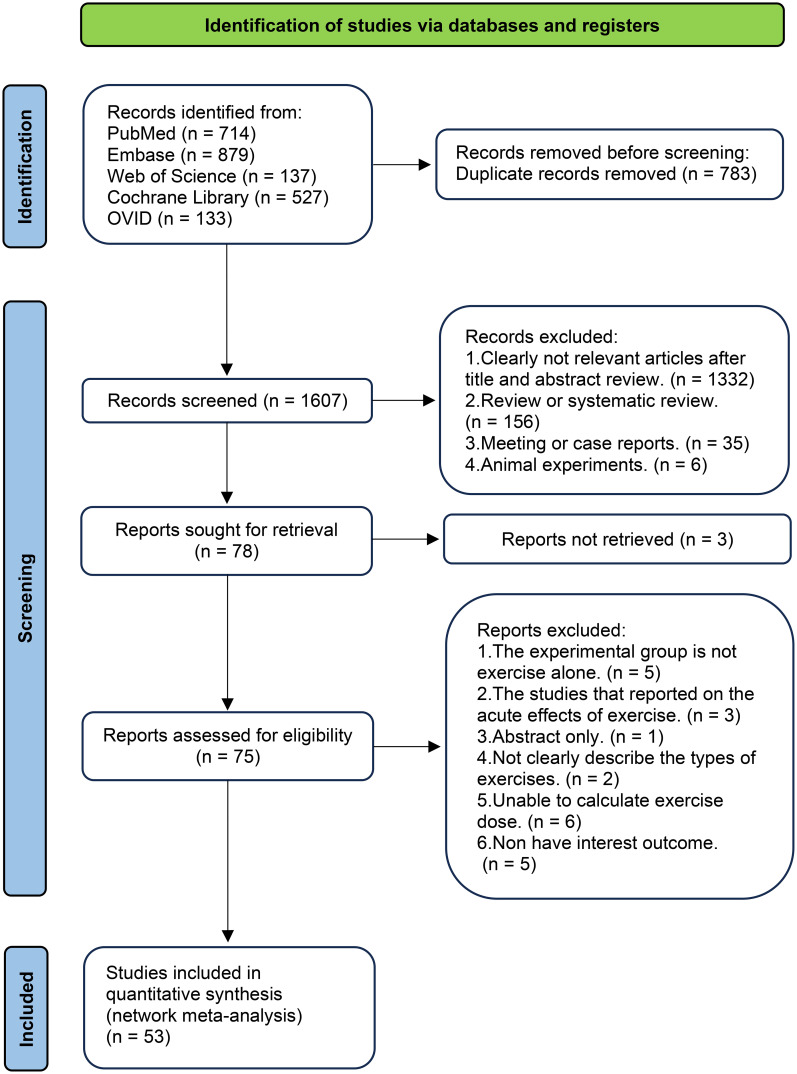
Flow diagram of literature selection.

A total of 2,390 records were initially retrieved from the electronic databases. After removal of duplicates, 1,607 records remained for title and abstract screening, of which 1,529 were excluded. The full texts of the remaining 78 articles were then assessed, and 25 were excluded for reasons such as inclusion of nonisolated exercise interventions, examination of acute rather than training effects, unclear intervention types, or insufficient information to calculate exercise dosage. Ultimately, 53 studies met all eligibility criteria and were included in the final analysis (**Figure 2**).

### 3.2 Quality assessment of the included studies

Only a subset of the included studies achieved simultaneous blinding of participants and outcome assessors. However, given that the intervention involved exercise training, implementing full blinding was inherently challenging, as both the participants and their guardians were required to provide informed consent prior to participation. Detailed assessments of blinding and other methodological characteristics are presented in **Supplementary Figure 1** and **S2**.

### 3.3 Characteristics of the included studies

In total, 53 RCTs involving 1,580 soccer players were included in this review. The interventions in the experimental groups comprised strength training (16 studies) (Chelly et al., 2009; Negra et al., 2016; Krommes et al., 2017; Katushabe and Kramer, 2020; Silva et al., 2022; Boraczyński M. et al., 2023; Durán-Custodio et al., 2023; Pecci et al., 2023; Torres Martín et al., 2023; Alsharab et al., 2024; Belamjahad et al., 2024; Ben Abderrahman et al., 2024; Darragi et al., 2024; Gołaś et al., 2024; Yang et al., 2024; Durán-Custodio et al., 2025), speed training (12 studies) (Sedano et al., 2011; Michailidis et al., 2013; Jastrzębski, 2014; Hammami et al., 2016; Coratella et al., 2018; Jlid et al., 2019; Aloui et al., 2022; Bianchi et al., 2023; Liu et al., 2024; Söyler et al., 2024; Sal-de-Rellán et al., 2024; Matuszczyk et al., 2025), agility training (11 studies) (Polman et al., 2004; Bloomfield et al., 2007; Jovanovic et al., 2011; Milanović et al., 2014; Trecroci et al., 2016; Padrón-Cabo et al., 2020; González-Fernández et al., 2021; Padrón-Cabo et al., 2021; Changyuan et al., 2024; Lee et al., 2024; Neag et al., 2025), and endurance training (14 studies) (Helgerud et al., 2001; Impellizzeri et al., 2006; Sperlich et al., 2011; Ingebrigtsen et al., 2013; Faude et al., 2014; Haugen et al., 2014; Los Arcos et al., 2015; Mackała et al., 2019; Arslan et al., 2020; Lesinski et al., 2021; Clemente et al., 2022; Nayıroğlu et al., 2022; Boraczyński MT. et al., 2023; Majkić et al., 2025). Among the included studies, 32 reported CMJ as an outcome measure, 18 reported SJ, 7 reported 1-RM squat, 19 reported 5m-sprint, 32 reported 10m-sprint, 27 reported 30m-sprint, 11 reported the T test, and 12 reported VO_2_max. Geographically, the studies were distributed as follows: three from East Asia, thirty-seven from Europe, six from the Middle East, one from Southeast Asia, and six from Africa. The detailed characteristics of the included studies are presented in **Supplementary Table 2**.

### 3.4 Network meta-analysis

**Figure 3:**
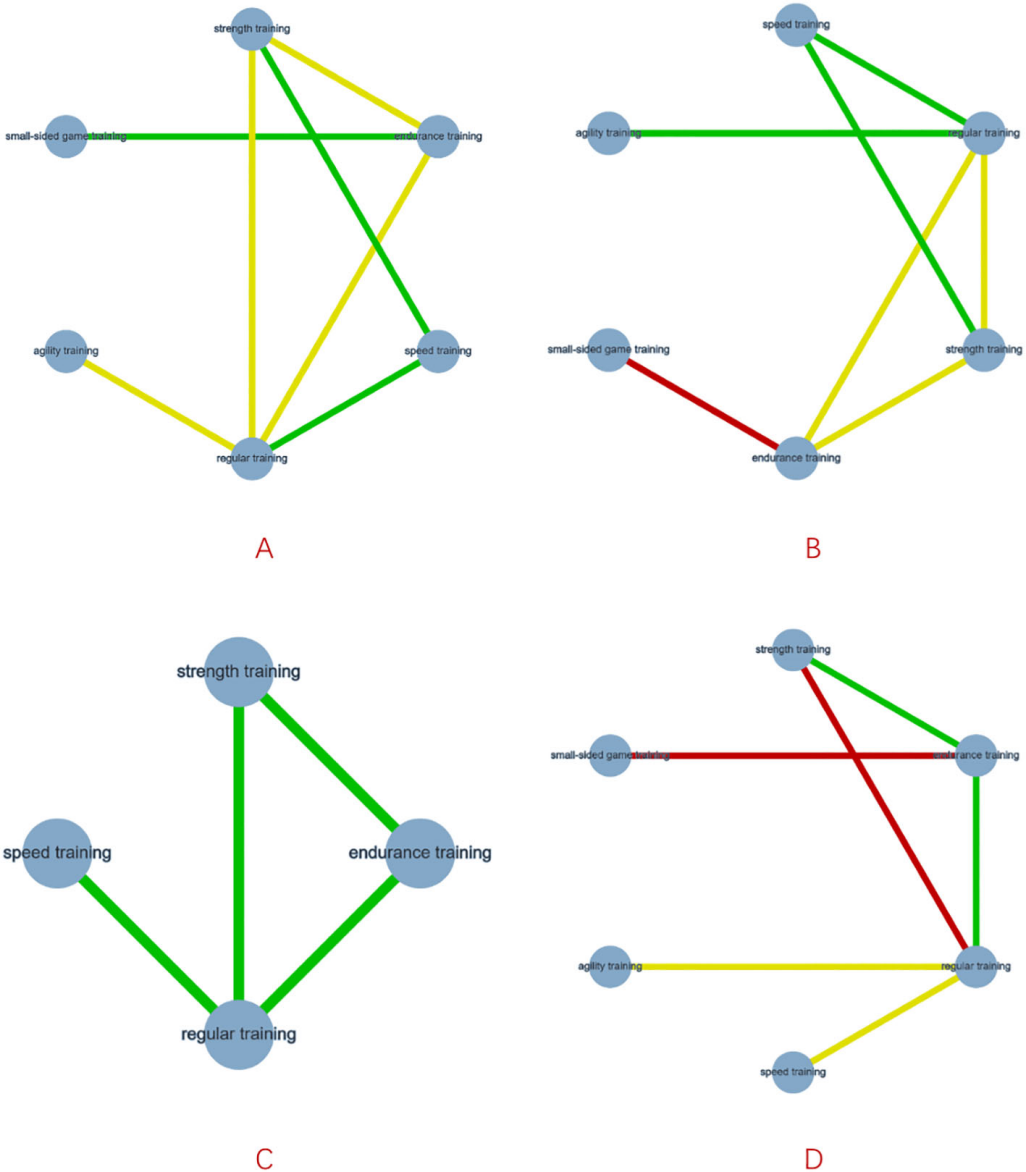
NMA figure. **(A)** CMJ; **(B)** SJ; **(C)** 1-RM squat; **(D)** 5-m sprint.

**Figure 4:**
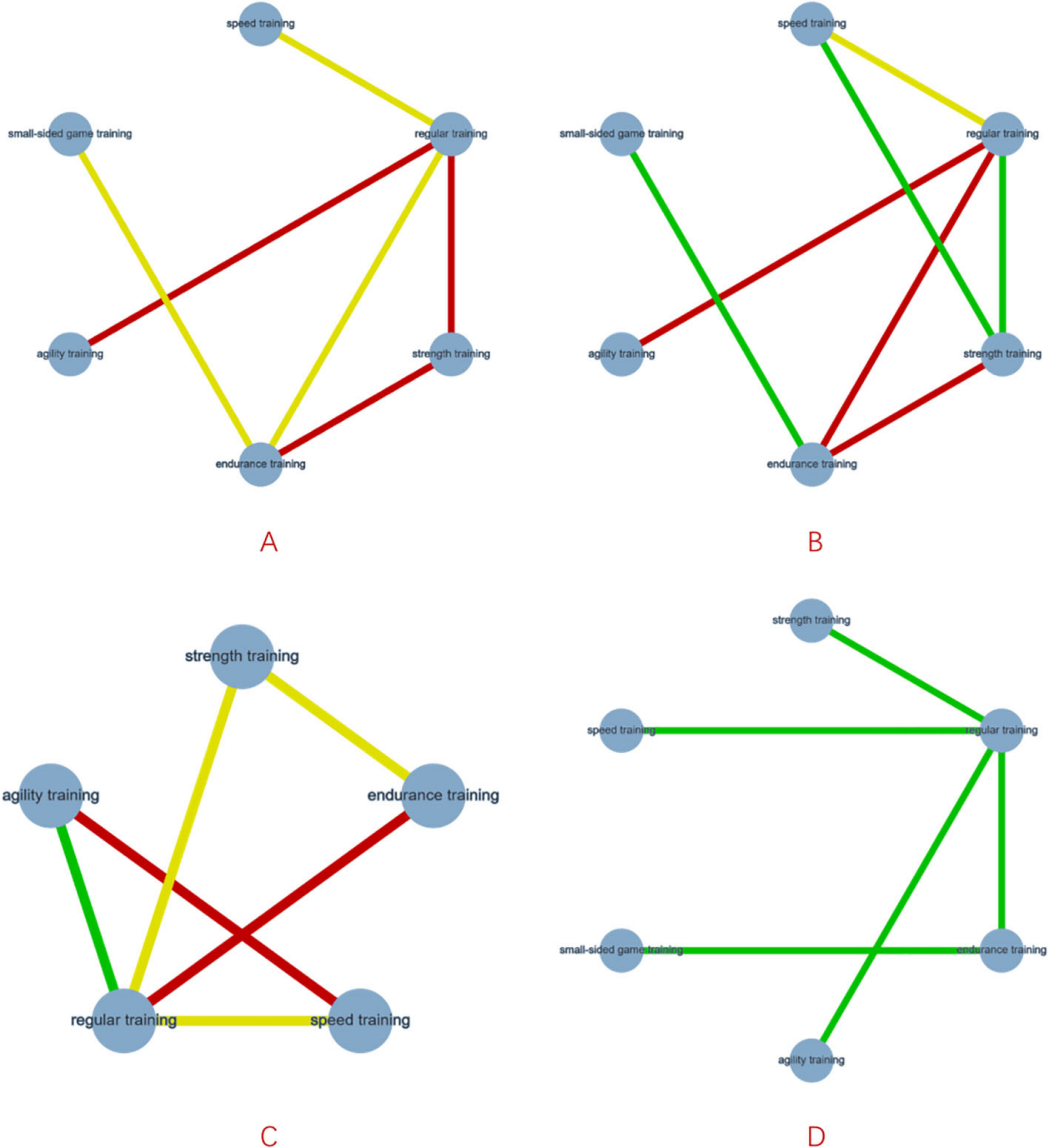
NMA figure. **(A)** 10-m sprint; **(B)** 30-m sprint; **(C)** T-test; **(D)** VO2max.

The complete NMA network diagrams and corresponding analytical results will be presented in **Figures 3**, **4**.

#### 3.4.1 CMJ

All P-values derived from the consistency and inconsistency assessments of indirect and direct comparisons across the included studies exceeded 0.05, indicating that the level of agreement between evidence sources was acceptable and that the assumption of consistency within the NMA framework was supported.

**Figure 5:**
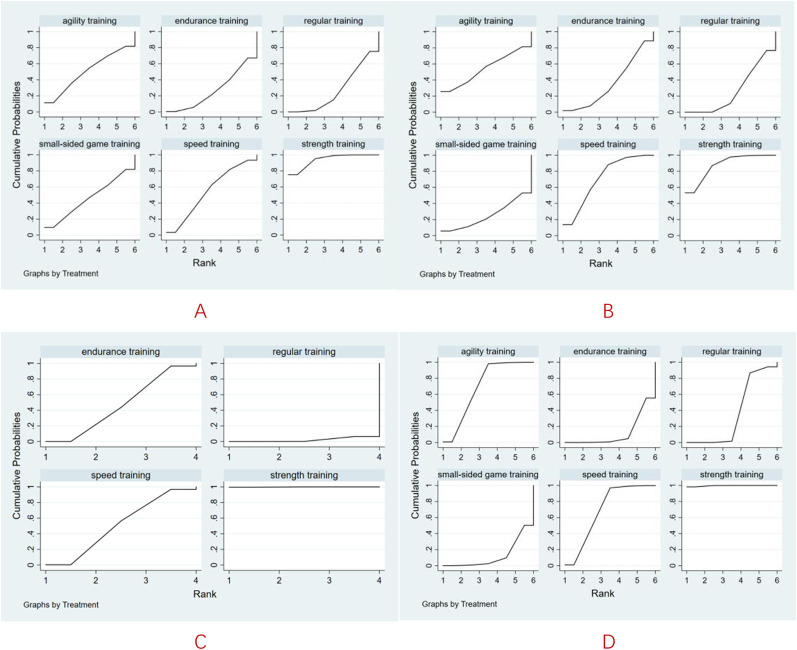
SUCRA plot. **(A)** CMJ; **(B)** SJ; **(C)** 1-RM squat; **(D)** 5-m sprint.

The results of the network meta-analysis indicated that, compared with the control group receiving regular training, strength training demonstrated a superior effect on CMJ performance [MD = 2.55, 95% CI = (1.17, 3.92)]. Additionally, strength training was more effective than endurance training [MD = 2.67, 95% CI = (0.14, 5.21)] in enhancing CMJ performance. According to the SUCRA-based probability rankings, strength training showed the highest likelihood of being the most effective intervention for improving CMJ performance (SUCRA: 94%), as illustrated in **Figure 5A**. Pairwise comparisons between the different interventions are presented in **Supplementary Table 3A**. A comparison between the two different interventions will be shown in **Supplementary Table 4A**.

#### 3.4.2 Squat jump

All P-values obtained from the assessments of consistency and inconsistency between indirect and direct comparisons in the included studies exceeded 0.05, indicating an acceptable level of agreement across the evidence and supporting the assumption of consistency within the NMA framework.

The network meta-analysis indicated that, compared with the control group receiving regular training, both strength training [MD = 2.81, 95% CI = (1.29, 4.32)] and speed training [MD = 2.06, 95% CI = (0.75, 3.38)] were more effective in enhancing SJ performance. According to the SUCRA-based probability rankings, strength training demonstrated the highest likelihood of being the most effective intervention for improving SJ performance (SUCRA: 87.5%), as illustrated in **Figure 5B**. Pairwise comparisons among the interventions are presented in **Supplementary Table 3B**. A comparison between the two different interventions will be shown in **Supplementary Table 4B**.

#### 3.4.3 One rep max squat

All P-values derived from the assessments of direct and indirect comparisons across studies exceeded 0.05, indicating satisfactory consistency within the network and supporting the validity of the NMA’s underlying consistency assumption.

The results of the network meta-analysis indicated that, compared with the control group receiving regular training, strength training was superior in improving 1-RM squat performance [MD = 31.23, 95% CI = (24.17, 38.3)]. Similarly, strength training outperformed endurance training [MD = 20.75, 95% CI = (10.8, 30.7)] and speed training [MD = 19.44, 95% CI = (5.21, 33.66)] in enhancing 1-RM squat performance. According to the SUCRA-based probability rankings, strength training demonstrated the highest likelihood of being the most effective intervention for improving 1-RM squat performance (SUCRA: 99.9%), as shown in **Figure 5C**. Pairwise comparisons between the interventions are presented in **Supplementary Table 3C**. A comparison between the two different interventions will be shown in **Supplementary Table 4C**.

#### 3.4.4 Five meters sprint

All P-values for the consistency and inconsistency tests between indirect and direct comparisons across the studies were greater than 0.05, indicating that the level of consistency between the studies was acceptable.

The results of the network meta-analysis demonstrated that, relative to the control group receiving regular training, strength training [MD = -0.09, 95% CI = (-0.12, -0.06)], agility training [MD = -0.04, 95% CI = (-0.07, -0.01)], and speed training [MD = -0.04, 95% CI = (-0.07, -0.00)] were more effective in improving 5-meter sprint performance. Compared to small-sided game training, strength training [MD = -0.13, 95% CI = (-0.19, -0.07)], agility training [MD = -0.08, 95% CI = (-0.14, -0.01)], and speed training [MD = -0.08, 95% CI = (-0.15, -0.01)] also showed superior effects. Additionally, relative to endurance training, strength training [MD = -0.13, 95% CI = (-0.18, -0.08)], agility training [MD = -0.07, 95% CI = (-0.13, -0.01)], and speed training [MD = -0.07, 95% CI = (-0.14, -0.01)] were more effective in improving 5-meter sprint performance. When compared with speed training, strength training [MD = -0.05, 95% CI = (-0.10, -0.01)] demonstrated superior results, and similarly, strength training [MD = -0.05, 95% CI = (-0.10, -0.01)] outperformed agility training in improving 5-meter sprint performance. According to the SUCRA-based probability rankings, strength training had the highest likelihood of being the most effective intervention for improving 5-meter sprint performance (SUCRA: 99.6%), as shown in **Figure 5D**. Pairwise comparisons between the interventions are presented in **Supplementary Table 3D**. A comparison between the two different interventions will be shown in **Supplementary Table 4D**.

#### 3.4.5 Ten meters sprint

All P-values for the consistency and inconsistency tests between the indirect and direct comparisons across the studies were greater than 0.05, indicating that the consistency between the studies was acceptable.

**Figure 6:**
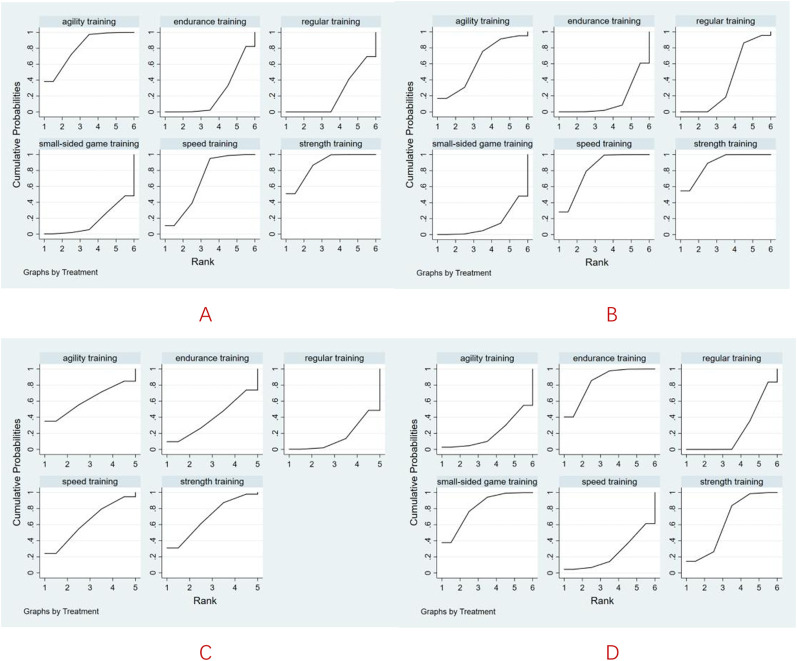
SUCRA plot. **(A)** 10-m sprint; **(B)** 30-m sprint; **(C)** T-test; **(D)** VO2max.

The results of the network meta-analysis indicated that, compared to the control group receiving regular training, strength training [MD = -0.09, 95% CI = (-0.13, -0.05)], agility training [MD = -0.09, 95% CI = (-0.14, -0.03)], and speed training [MD = -0.07, 95% CI = (-0.11, -0.03)] were more effective in improving 10-meter sprint performance. Similarly, relative to endurance training, strength training [MD = -0.09, 95% CI = (-0.15, -0.04)], agility training [MD = -0.09, 95% CI = (-0.16, -0.01)], and speed training [MD = -0.07, 95% CI = (-0.14, -0.00)] were superior in enhancing 10-meter sprint performance. When compared to small-sided game training, strength training [MD = -0.10, 95% CI = (-0.19, -0.02)] also showed better results. According to the SUCRA-based probability rankings, strength training demonstrated the highest likelihood of being the most effective intervention for improving 10-meter sprint performance (SUCRA: 87.4%), as shown in **Figure 6A**. Pairwise comparisons between the interventions are presented in **Supplementary Table 3E**. A comparison between the two different interventions will be shown in **Supplementary Table 4E**.

#### 3.4.6 Thirty meters sprint

All P-values for the consistency and inconsistency tests between indirect and direct comparisons across the studies were greater than 0.05, indicating that the consistency between studies was acceptable.

The results of the network meta-analysis indicated that, relative to the control group receiving regular training, both strength training [MD = -0.18, 95% CI = (-0.27, -0.09)] and speed training [MD = -0.15, 95% CI = (-0.24, -0.07)] were superior in improving 30-meter sprint performance. When compared to endurance training, strength training [MD = -0.30, 95% CI = (-0.47, -0.12)] and speed training [MD = -0.27, 95% CI = (-0.46, -0.08)] also demonstrated superior effects. Additionally, compared to small-sided game training, both strength training [MD = -0.30, 95% CI = (-0.53, -0.08)] and speed training [MD = -0.28, 95% CI = (-0.51, -0.05)] outperformed in enhancing 30-meter sprint performance. According to the SUCRA-based probability rankings, strength training exhibited the highest likelihood of being the most effective intervention for improving 30-meter sprint performance (SUCRA: 88.8%), as shown in **Figure 6B**. Pairwise comparisons between the interventions are presented in **Supplementary Table 3F**. A comparison between the two different interventions will be shown in **Supplementary Table 4F**.

#### 3.4.7 T-test for agility

All P-values for the consistency and inconsistency tests between indirect and direct comparisons across the studies exceeded 0.05, indicating that the consistency between studies was acceptable.

The probability ranking of various exercise interventions for improving T-test performance indicated that strength training ranked first in the SUCRA (SUCRA: 69.4%), as shown in **Figure 6C**. Pairwise comparisons between the different interventions are presented in **Supplementary Table 3G**. A comparison between the two different interventions will be shown in **Supplementary Table 4G**.

#### 3.4.8 Maximal oxygen uptake (VO2max)

All P-values for the consistency and inconsistency tests between indirect and direct comparisons across the studies were greater than 0.05, indicating that the consistency between studies was deemed acceptable.

The results of the network meta-analysis demonstrated that, relative to the control group receiving regular training, endurance training [MD = 1.94, 95% CI = (0.79, 3.09)], small-sided game training [MD = 1.90, 95% CI = (0.54, 3.27)], and strength training [MD = 1.34, 95% CI = (0.52, 2.16)] were more effective in improving VO_2_max performance. According to the SUCRA-based probability rankings, endurance training showed the highest likelihood of being the most effective intervention for improving VO_2_max performance (SUCRA: 84.7%), as shown in **Figure 6D**. Pairwise comparisons between the interventions are presented in **Supplementary Table 3H**. A comparison between the two different interventions will be shown in **Supplementary Table 4H**.

### 3.5 Dose–response relationship

#### 3.5.1 CMJ

**Figure 7:**
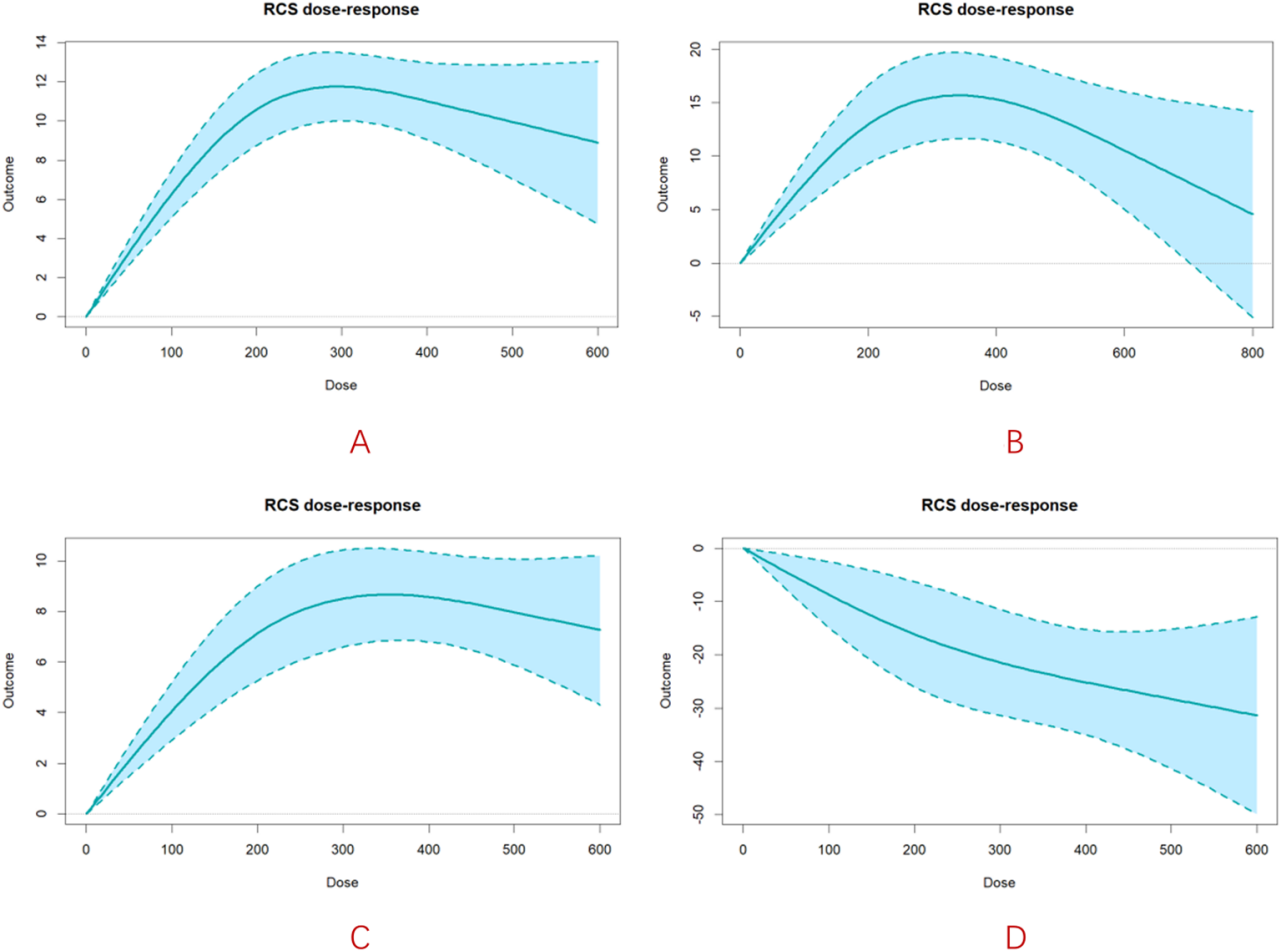
Dose-response association between overall exercise dose and change. **(A)** CMJ; **(B)** SJ; **(C)** 1-RM squat; **(D)** 5-m sprint.

We plotted the non-linear dose-response relationship between overall exercise dosage and CMJ performance using the ggplot2 package in R (**Figure 7A**). A U-shaped relationship was observed between the overall exercise dose and CMJ performance, with the optimal dose peak identified. The optimal exercise dose was estimated at 296.97 METs-min/week (SMD: 11.753, 95% CI: 10.005 to 13.502) for improving overall performance. Prior to reaching the optimal dose peak, each additional unit of dose resulted in an average increase of 0.041 in CMJ performance. However, once beyond the peak, each additional unit of dose resulted in an average decrease of 0.01 in CMJ performance.

#### 3.5.2 Squat jump

We plotted the non-linear dose-response relationship between overall exercise dosage and SJ performance using the ggplot2 package in R (**Figure 7B**). A U-shaped relationship was observed between exercise dosage and SJ performance, with the optimal dose peak identified. The optimal exercise dose was estimated at 339.39 METs-min/week (SMD: 15.694, 95% CI: 11.658 to 19.73) for improving overall performance. Before reaching the peak optimal dose, each additional unit of dose resulted in an average increase of 0.048 in SJ performance. However, after surpassing the optimal dose peak, each additional unit of dose led to an average decrease of 0.026 in SJ performance.

#### 3.5.3 One rep max squat

We plotted the non-linear dose-response relationship between overall exercise dosage and 1-RM squat performance using the ggplot2 package in R (**Figure 7C**). A U-shaped relationship was observed between exercise dosage and 1-RM squat performance, with the optimal dose peak identified. The optimal exercise dose was estimated at 351.52 METs-min/week (SMD: 8.66, 95% CI: 6.843 to 10.478) for improving overall performance. Prior to reaching the optimal dose peak, each additional unit of dose resulted in an average increase of 0.025 in 1-RM squat performance. However, after surpassing the peak of the optimal dose, each additional unit of dose led to an average decrease of 0.006 in 1-RM squat performance.

#### 3.5.4 Five meters sprint

We plotted the linear dose-response relationship between overall exercise dosage and 5m-sprint performance using the ggplot2 package in R (**Figure 7D**). A linear relationship was observed, with 5m-sprint performance gradually decreasing as the exercise dose increased. The analysis revealed that within the 0–600 dose range, each additional unit of dose led to an average reduction of 0.05 in 5m-sprint performance.

#### 3.5.5 Ten meters sprint

**Figure 8:**
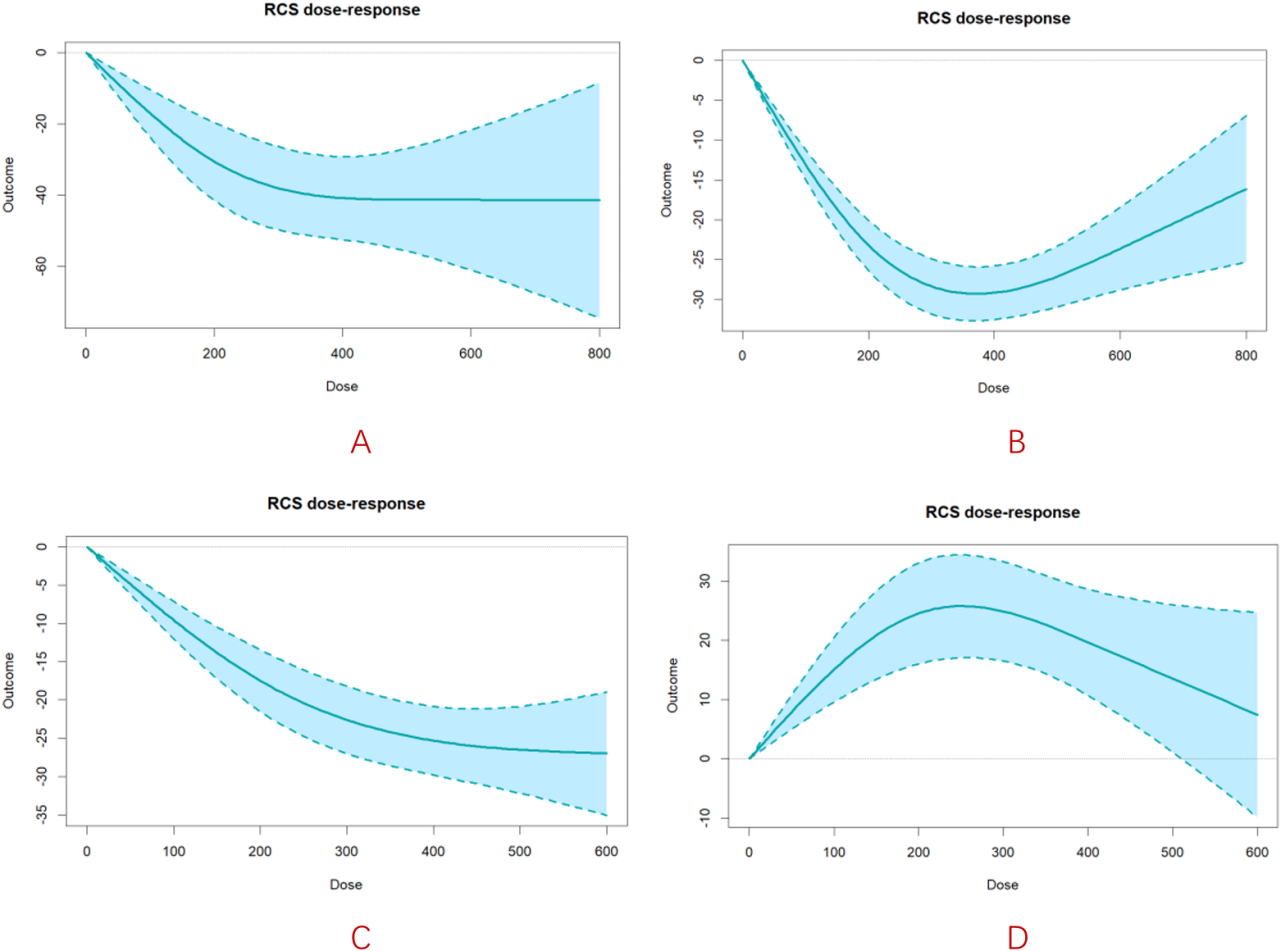
Dose-response association between overall exercise dose and change. **(A)** 10-m sprint; **(B)** 30-m sprint; **(C)** T-test; **(D)** VO2max.

We plotted the linear dose-response relationship between overall exercise dosage and 10m-sprint performance using the ggplot2 package in R (**Figure 8A**). A linear relationship was observed, with 10m-sprint performance gradually decreasing as the exercise dose increased. The analysis showed that within the 0–800 dose range, each additional unit of dose resulted in an average reduction of 0.04 in 10m-sprint performance.

#### 3.5.6 Thirty meters sprint

We plotted the non-linear dose-response relationship between overall exercise dosage and 30m-sprint performance using the ggplot2 package in R (**Figure 8B**). A U-shaped relationship was observed between exercise dosage and 30m-sprint performance, with the optimal dose peak identified. The optimal exercise dose was estimated at 371.72 METs-min/week (SMD: -29.274, 95% CI: -32.657 to -25.891) for improving overall performance. Before reaching the optimal dose peak, each additional unit of dose resulted in an average decrease of 0.081 in 30m-sprint performance. After surpassing the peak, each additional unit of dose led to an average increase of 0.033 in 30m-sprint performance.

#### 3.5.7 T-test for agility

We plotted the linear dose-response relationship between overall exercise dosage and T-test performance using the ggplot2 package in R (**Figure 8C**). A linear relationship was observed, with T-test performance gradually decreasing as the exercise dose increased. The analysis showed that within the 0–600 dose range, each additional unit of dose resulted in an average reduction of 0.043 in T-test performance.

#### 3.5.8 Maximal oxygen uptake (VO2max)

We plotted the non-linear dose-response relationship between overall exercise dosage and VO_2_max performance using the ggplot2 package in R (**Figure 8D**). A U-shaped association was observed between exercise dosage and VO_2_max performance, allowing identification of the optimal dose peak. The optimal exercise dose was estimated at 248.48 METs-min/week (SMD: 25.764, 95% CI: 17.063 to 34.466) for maximizing overall performance. Before reaching this optimal dose, each additional unit of exercise dosage resulted in an average increase of 0.108 in VO_2_max. Conversely, after surpassing the optimal dose peak, each additional unit of dosage produced an average decrease of 0.056 in VO_2_max.

### 3.6 Publication bias test

**Figure 9:**
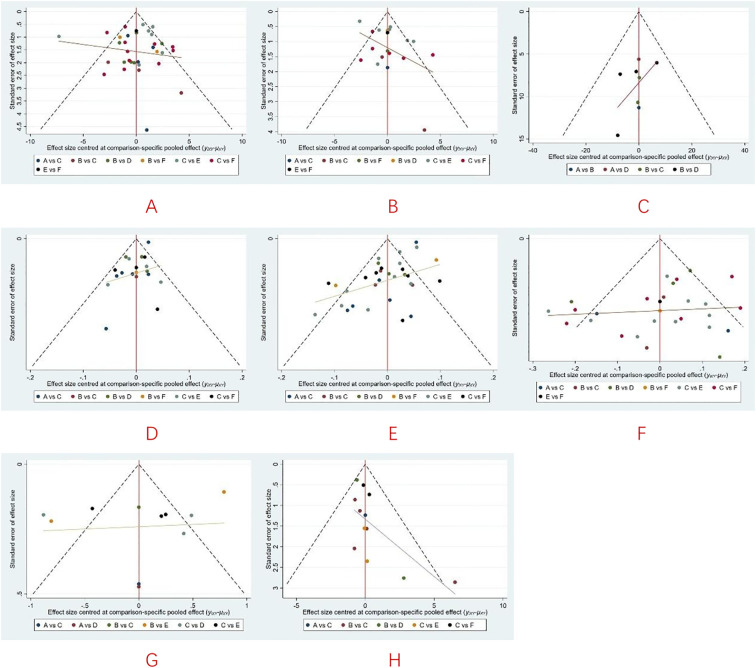
Funnel plot on publication bias. **(A)** CMJ; **(B)** SJ; **(C)** 1-RM squat; **(D)** 5-m sprint; **(E)** 10-m sprint; **(F)** 30-m sprint; **(G)** T-test; **(H)** VO2max.

Separate funnel plots were constructed for each outcome indicator to examine the potential influence of publication bias. The visual symmetry observed across the plots suggests that publication bias was unlikely to have affected the results. Detailed depictions of these analyses are shown in **Figure 9**.

## 4 Discussion

In this study, we compared the effectiveness of various physical training interventions on enhancing the athletic performance of soccer players and examined the optimal training dosage across different exercise modalities. A total of 53 studies were included, encompassing four distinct training approaches and involving 1,580 soccer players, thereby providing a robust sample size. Our findings indicate that strength training is the most effective intervention for improving CMJ and SJ performance, as well as for increasing 1-RM squat maximal strength. Strength training also produced the greatest reductions in 5 m-sprint, 10 m-sprint, 30 m-sprint, and T-test completion times. In contrast, endurance training demonstrated superior benefits in enhancing VO_2_max. Furthermore, we identified a U-shaped relationship between total training dosage and performance outcomes in CMJ, SJ, 1-RM squat, 30 m-sprint, and VO_2_max, and subsequently determined the optimal dosage thresholds for these indicators. Overall, although different training modalities show specific advantages, our results suggest that strength training may represent the most suitable intervention for improving the comprehensive athletic performance of soccer players.

Strength training has been shown to produce substantial improvements in the athletic performance of soccer players by enhancing force transmission efficiency, optimizing sport-specific physical qualities, reducing injury risk, and supporting sustained competitive capacity (Styles et al., 2016; Zouita et al., 2016). As simple, equipment-free assessments of explosive power, CMJ and SJ are widely used in performance monitoring among soccer players. The Fédération Internationale de Football Association (FIFA) Physical Fitness Tests Guidelines recommend incorporating the difference between CMJ and SJ—representing elastic energy contribution—into the evaluation system. Athletes with larger CMJ–SJ differences typically exhibit superior performance in positions requiring rapid explosive actions (e.g., forward, fullbacks), whereas those with smaller differences may benefit from targeted elastic muscle training, such as depth jumps (Roderick, 2012). Additionally, the fatigue index derived from ten consecutive CMJ trials provides an indicator of lower-limb strength endurance, which is negatively correlated with high-intensity running distance during the latter stages of competition, thereby offering valuable reference for endurance-specific conditioning. Strength training refers to a systematic training approach that induces muscle contraction through external resistance to enhance muscular strength, explosive power, and neuromuscular coordination (Earle, 2008). Resistance stimuli elicit adaptive changes in muscle fibers—including hypertrophy, increased glycogen storage, and improved neural activation efficiency—which collectively contribute to optimized muscle function (Pillitteri et al., 2023). For soccer athletes, strength training extends beyond traditional hypertrophy-focused paradigms and instead emphasizes a functional training framework. This approach integrates strength development with technical movement patterns, targeting muscle groups essential for soccer-specific actions such as propulsion during sprinting, stabilization during physical duels, and force generation during shooting (Hoff and Helgerud, 2004). Functional strength programs therefore prioritize force application and transfer under dynamic and unstable conditions, ensuring direct translation to match performance (Gorostiaga et al., 2005). This characteristic underscores the need for multidimensional training designs that extend beyond single-load training models. Our findings demonstrate that strength training yields statistically significant improvements in maximal strength, jumping performance, linear sprinting ability, and agility among soccer players, with clear advantages over control conditions. These results are consistent with previous research and further support the efficacy of strength-based interventions in soccer performance enhancement.

Endurance training represents a fundamental component of soccer-specific conditioning and exerts comprehensive and profound benefits on athletic performance (Tønnessen et al., 2012). Its effects extend beyond improving an athlete’s capacity to sustain high-intensity activity, contributing also to the optimization of multiple physiological systems that support technical execution, tactical decision-making, and recovery from fatigue (Sander et al., 2013; Bedoya et al., 2015). As a key indicator of aerobic metabolic capacity, VO_2_max directly influences a soccer player’s ability to sustain repeated high-intensity efforts, maintain effective recovery between actions, and execute tactical strategies throughout competition (Helgerud et al., 2001). Soccer is characterized by a “high-intensity intermittent + prolonged duration” activity profile, with players typically covering 10–13 km per match. Of this distance, approximately 15%–20% consists of high-intensity sprints and direction changes, while 60%–70% involves low-intensity jogging or walking, demonstrating the sport’s substantial dependence on aerobic metabolism (Rampinini et al., 2007; Harper et al., 2019; Sarmento et al., 2024). Improvements in VO_2_max achieved through endurance training are not limited to increases in its absolute value; more importantly, they reflect enhanced coordination between oxygen transport and oxygen utilization systems, enabling a more precise alignment between aerobic capacity and the physiological demands of soccer (Sarmento et al., 2024). The advantages of such adaptations span multiple domains, including improved ability to sustain high workloads, accelerated fatigue recovery, enhanced tactical performance, reduced injury risk, and prolonged athletic career longevity (Malamud and Smukas, 2025). Consequently, endurance training remains a cornerstone of long-term performance development in soccer athletes. Our results indicate that endurance training produces statistically significant improvements in VO_2_max among soccer players compared with control groups, consistent with findings from previous studies.

Through systematic review and network meta-analysis, this study elucidated the differentiated associations between training dosage and eight key performance indicators in soccer players, providing essential evidence for the precise design of sport-specific conditioning programs. The dosage-related findings highlight two overarching principles: quality-specific physiological responses and the inherent trade-off between training dosage and performance. Among the observed patterns, five indicators—CMJ, SJ, 1-RM squat, 30 m-sprint, and VO_2_max—exhibited U-shaped dose–response relationships, with optimal dosages spanning a relatively wide range (248.48–371.72 METs·min/week). VO_2_max demonstrated the highest sensitivity to training stimuli, showing the lowest optimal dosage and the greatest improvement efficiency during low-dose training (0.108 per unit dosage), suggesting that aerobic endurance training should avoid excessive loading. In contrast, the 30 m-sprint, characterized as a speed–endurance composite indicator, required the highest optimal dosage and yielded the largest low-dose improvement (0.081 per unit dosage), indicating that mid-distance sprint performance relies heavily on sufficient training volume. The 1-RM squat demonstrated the greatest tolerance to suprathreshold training, with only a minimal decline observed beyond the optimal dosage (a reduction of 0.006 per unit dosage), reflecting the relative stability of strength development. These variations are attributable to the distinct physiological underpinnings of each performance component: aerobic endurance depends on mitochondrial adaptations, explosive power on neuromuscular coordination, and maximal strength on muscle hypertrophy—each characterized by a unique “load threshold” and “tolerance ceiling.” Notably, the 5 m-sprint, 10 m-sprint, and T-test exhibited linear negative associations with training dosage, lacking identifiable optimal dosages and showing continuous performance decline with increasing load. This phenomenon underscores a fundamental conflict inherent in soccer’s multifaceted physical demands: short-distance sprinting and agility rely on fast-twitch muscle fiber explosiveness and rapid neural activation, whereas excessive physical conditioning—particularly aerobic-oriented training—may induce fiber-type shifts, accumulate neuromuscular fatigue, and subsequently impair acceleration and change-of-direction ability. These findings hold practical implications for training design. For example, forward should maintain strict control over total training load to preserve short-distance sprint capacity, whereas midfielders must balance the optimal dosage ranges for VO_2_max and 30 m-sprint performance to achieve comprehensive performance enhancement.

Overall, the findings of this study clearly refute the traditional assumption that higher training dosages necessarily yield superior outcomes. Instead, the results underscore the importance of adhering to the principle of appropriate loading in the physical conditioning of soccer players. Training dosage should be precisely prescribed according to the physiological characteristics of each performance indicator and dynamically adjusted based on positional demands. Moreover, strategies such as phased training, low-dose high-frequency scheduling, and targeted load distribution are essential for mitigating the inherent conflicts between different fitness components. Through such tailored approaches, comprehensive enhancement of athletic performance can be effectively achieved.

## 5 Strengths and limitations

First, our study included 53 investigations encompassing a total of 1,580 soccer players, providing a robust and substantial sample size. In addition, building upon existing reviews examining the effects of physical training on athletic performance, we incorporated multiple intervention modalities and conducted direct comparisons among them. This approach enabled us to generate updated and more comprehensive evidence-based recommendations for optimizing training strategies.

Secondly, our study inherits certain limitations from the original research on which it is based. Although we made every effort to minimize heterogeneity when selecting and analyzing the included studies, some degree of variability was unavoidable—for example, differences in regional distribution and the proportion of male and female participants across studies.

Finally, readers should interpret the findings with caution, as several interventions were represented by a relatively small number of studies and limited head-to-head comparative evidence. These gaps underscore the need for further high-quality research to expand the evidence base and strengthen the reliability of future conclusions.

## 6 Conclusions

This study suggests that soccer players aiming to improve explosive power, linear sprint performance, and agility should prioritize targeted strength training. Conversely, athletes seeking to enhance aerobic metabolic capacity may benefit more from endurance-focused training. Importantly, for players pursuing comprehensive improvements in overall athletic performance, the most effective approach appears to be a concurrent training model that integrates both strength and endurance training. Such a combined strategy facilitates the coordinated development of multiple physical qualities and provides a robust foundation for enhancing overall competitive performance.

## Data Availability

The datasets presented in this article are not readily available. Requests to access the datasets should be directed to Shuyuan Du, dsybsu@126.com.
