# Supplementary material for: The optimal dose of strength and conditioning training for enhancing physical performance in football players: a systematic review and network meta-analysis of randomized clinical trials
Source: Front Physiol. 2026 Jul 6;17:1791257. doi: 10.3389/fphys.2026.1791257 (PMC13381195; doi:10.3389/fphys.2026.1791257)
Supplement: Supplementary file 1 [file DataSheet1.docx]

Table S1. Search strategy on PubMed.

| #1 | ((((((((((Football[MeSH Terms]) OR (Soccer[MeSH Terms])) OR (Football[Title/Abstract])) OR (Soccer[Title/Abstract])) OR (American Football[Title/Abstract])) OR (Football, American[Title/Abstract])) OR (Football European[Title/Abstract])) OR (European, Football[Title/Abstract])) OR (Europeans, Football[Title/Abstract])) OR (European Football[Title/Abstract])) OR (Football, European[Title/Abstract]) |
| --- | --- |
| #2 | (((((((((((((((((((((((((((((((((((((((((((((((Physical Education and Training[MeSH Terms]) OR (Physical Conditioning, Human[MeSH Terms])) OR (Exercise[MeSH Terms])) OR (Physical Education[Title/Abstract] AND Training[Title/Abstract])) OR (Physical Conditioning, Human[Title/Abstract])) OR (Exercise[Title/Abstract])) OR (Physical Education, Training[Title/Abstract])) OR (Physical Education[Title/Abstract])) OR (Education, Physical[Title/Abstract])) OR (Conditioning, Human Physical[Title/Abstract])) OR (Human Physical Conditioning[Title/Abstract])) OR (Physical Training, Human[Title/Abstract])) OR (Human Physical Training[Title/Abstract])) OR (Training, Human Physical[Title/Abstract])) OR (Physical Exercise[Title/Abstract])) OR (Isometric Exercises[Title/Abstract])) OR (Exercise Training[Title/Abstract])) OR (Physical Activities[Title/Abstract])) OR (Training[Title/Abstract])) OR (Intervention[Title/Abstract])) OR (strength[Title/Abstract])) OR (power[Title/Abstract])) OR (explosive[Title/Abstract])) OR (resistance[Title/Abstract])) OR (velocity-based resistance[Title/Abstract])) OR (ballistic[Title/Abstract])) OR (plyometric[Title/Abstract])) OR (offset load[Title/Abstract])) OR (TRX[Title/Abstract])) OR (Speed[Title/Abstract])) OR (Jump[Title/Abstract])) OR (linear speed[Title/Abstract])) OR (multidirectional speed[Title/Abstract])) OR (acceleration-speed[Title/Abstract])) OR (reaction speed[Title/Abstract])) OR (running economy[Title/Abstract])) OR (endurance[Title/Abstract])) OR (high-intensity interval training[Title/Abstract])) OR (moderate-intensity continuous training[Title/Abstract])) OR (long slow distance[Title/Abstract])) OR (coordination[Title/Abstract])) OR (flexibility[Title/Abstract])) OR (static stretching[Title/Abstract])) OR (dynamic stretching[Title/Abstract])) OR (proprioceptive neuromuscular facilitation[Title/Abstract])) OR (agility[Title/Abstract])) OR (multimodal agility-based exercise training[Title/Abstract])) OR (SAQ[Title/Abstract]) |
| #3 | #1 AND #2 |
| #4 | (((randomized controlled[Title/Abstract]) OR (random[Title/Abstract])) OR (RCT[Title/Abstract])) OR (clinical trial[Title/Abstract]) |
| #5 | #3 AND #4 |


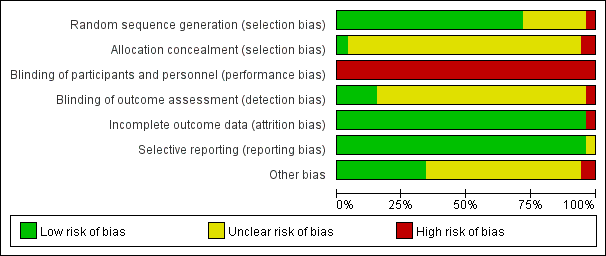


Figure S1. Summary of methodological qualities of included studies.


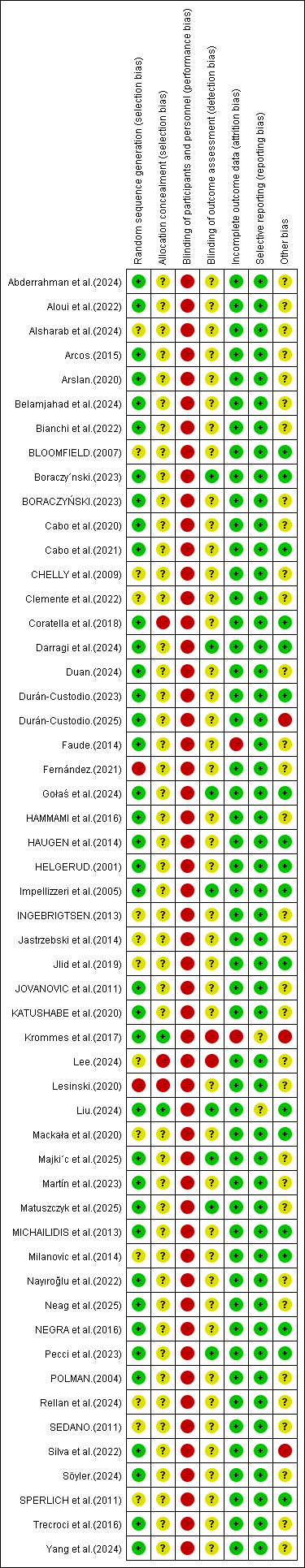


Figure S2. The result of risk of bias.

Table S2. Characteristics of the studies included in the meta-analysis.

| **Author** | **Country** | **Year** | **Population** | **Age**  **(Mean+SD)** | **Total/male/female** | **Intervention** | **Control** | **Outcome** |
| --- | --- | --- | --- | --- | --- | --- | --- | --- |
| Aloui | Tunisia | 2022 | Soccer players | T:14.6(0.5)  C: 14.6(0.4) | T:17/17/0  C:17/17/0 | Speed training  Length of Intervention: 8 weeks  Freq: Five times a week  Duration: 90min | CON | SJ, 10m sprint, 30m sprint |
| Belamjahad | France | 2024 | Soccer players | T:17(1.3)  C: 17(1.6) | T:12/0/12  C:12/0/12 | Strength training  Length of Intervention: 6 weeks  Freq: Three times a week  Duration: 60min | CON | CMJ, SJ, 1-RM squat, 5m sprint, 10m sprint, 30m sprint, T test |
| Abderrahman | Tunisia | 2024 | Soccer players | T+C:21.8(2.6) | T:15/15/0  C:15/15/0 | Strength training  Length of Intervention: 8 weeks  Freq: Five times a week  Duration: 90min | CON | CMJ, SJ, 30m sprint |
| Bianchi | UK | 2022 | Soccer players | T+C:20(2) | T:10/10/0  C:10/10/0 | Speed training  Length of Intervention: 6 weeks  Freq: Twice a week  Duration: 60min | CON | 10m sprint, 30m sprint |
| Boraczy´nski | Poland | 2023 | Soccer players | T:22.1(3.15)  C:22.1(4.21) | T:15/15/0  C:17/17/0 | Strength training  Length of Intervention: 8 weeks  Freq: Twice a week  Duration: 60min | CON | CMJ, SJ, 1-RM squat, 10m sprint, 30m sprint |
| Coratella | Italy | 2018 | Soccer players | T+C:21(3) | T:16/16/0  C:16/16/0 | Speed training  Length of Intervention: 8 weeks  Freq: Three times a week  Duration: 90min | CON | CMJ, SJ, 1-RM squat, 10m sprint, 30m sprint, T test |
| Darragi | Tunisia | 2024 | Soccer players | T:15.2(0.9)  C:15.5(0.9) | T:14/0/14  C:16/0/16 | Strength training  Length of Intervention: 12 weeks  Freq: Five times a week  Duration: 90min | CON | CMJ, SJ, 5m sprint, 10m sprint, 30m sprint, T test |
| Custodio | Spain | 2023 | Soccer players | T:24.5(3.1)  C: 25.8(2) | T:10/10/0  C:10/10/0 | Strength training  Length of Intervention: 12 weeks  Freq: Twice a week  Duration: 90min | CON | CMJ, SJ, 10m sprint |
| Castillo | Spain | 2025 | Soccer players | T:17.5(0.7)  C:17.7(0.5) | T:10/10/0  C:10/10/0 | Strength training  Length of Intervention: 12 weeks  Freq: Twice a week  Duration: 90min | CON | CMJ, SJ, 10m sprint |
| Gołaś | Poland | 2024 | Soccer players | T+C:25.8(7.3) | T:16/NA/NA  C:16/NA/NA | Strength training  Length of Intervention: 8 weeks  Freq: Four times a week  Duration: 90min | CON | 5m sprint, 30m sprint |
| Yang | China | 2024 | Soccer players | T:21.2(1.4)  C:22.2(1.9) | T:10/10/0  C:10/10/0 | Speed training  Length of Intervention: 6 weeks  Freq: Three times a week  Duration: 90min | CON | CMJ |
| Liu | Thailand | 2024 | Soccer players | T+C:16.3(0.6) | T:17/17/0  C:17/17/0 | Speed training  Length of Intervention: 9 weeks  Freq: Four times a week  Duration: 90min | CON | CMJ, SJ, 10m sprint |
| KATUSHABE | Africa | 2020 | Soccer players | T+C:20.47(1.85) | T:9/9/0  C:8/8/0 | Strength training  Length of Intervention: 6 weeks  Freq: Three times a week  Duration: 90min | CON | 1-RM squat |
| Majki´c | Serbia | 2025 | Soccer players | T:8.91(1.14)  C: 8.73(1.01) | T:11/11/0  C:11/11/0 | Endurance training  Length of Intervention: 6 weeks  Freq: Five times a week  Duration: 90min | CON | 5m sprint, 10m sprint, 30m sprint, T test |
| Matuszczyk | Poland | 2025 | Soccer players | T+C:22.45(3.67) | T:10/10/0  C:10/10/0 | Speed training  Length of Intervention: 10 weeks  Freq: Four times a week  Duration: 90min | CON | 5m sprint, 30m sprint |
| Söyler | Turkey | 2024 | Soccer players | T:16.64(0.5)  C: 16.71(0.47) | T:14/14/0  C:14/14/0 | Speed training  Length of Intervention: 8 weeks  Freq: Three times a week  Duration: 60min | CON | 10m sprint, 30m sprint |
| Duan | China | 2024 | Soccer players | T:16.3(0.8)  C:15.9(0.9) | T:28/28/0  C:28/28/0 | Agility training  Length of Intervention: 8 weeks  Freq: Twice a week  Duration: 30min | CON | T test |
| Silva | Portugal | 2022 | Soccer players | T:20.3(0.6)  C:20.4(0.7) | T:9/9/0  C:9/9/0 | Strength training  Length of Intervention: 8 weeks  Freq: Three times a week  Duration: 60min | CON | 10m sprint, 30m sprint, VO2max |
| Martín | Spain | 2021 | Soccer players | T+C:15.6(0.5) | T:20/20/0  C:26/26/0 | Strength training  Length of Intervention: 15 weeks  Freq: Three times a week  Duration: 60min | CON | CMJ, 10m sprint, 30m sprint |
| Pecci | Spain | 2022 | Soccer players | T:20.8(2.6)  C:20.1(2.6) | T:12/12/0  C:12/12/0 | Strength training  Length of Intervention: 6 weeks  Freq: Twice a week  Duration: 60min | CON | CMJ |
| HELGERUD | Norway | 2001 | Soccer players | T+C:18.1(0.8) | T:9/9/0  C:10/10/0 | Endurance training  Length of Intervention: 8 weeks  Freq: Twice a week  Duration: 30min | CON | 1-RM squat, 10m sprint, VO2max |
| Negra | Tunisia | 2016 | Soccer players | T:12.8(0.2)  C:12.7(0.3) | T:13/13/0  C:11/11/0 | Strength training  Length of Intervention: 12 weeks  Freq: Five times a week  Duration: 90min | CON | CMJ, SJ, 1-RM squat, 5m sprint, 10m sprint, 30m sprint, T test |
| Krommes | Denmark | 2017 | Soccer players | T:23(3.9)  C:25.1(4.9) | T:9/9/0  C:10/10/0 | Strength training  Length of Intervention: 10 weeks  Freq: Three times a week  Duration: 60min | CON | CMJ, 5m sprint, 10m sprint, 30m sprint |
| Chelly | Tunisia | 2009 | Soccer players | T:17(0.3)  C:17(0.5) | T:11/11/0  C:11/11/0 | Strength training  Length of Intervention: 8 weeks  Freq: Twice a week  Duration: 120min | CON | CMJ, SJ, 1-RM squat |
| Lee | South Korea | 2024 | Soccer players | T:19(0.86)  C:18.8(0.78) | T:9/0/9  C:10/0/10 | Agility training  Length of Intervention: 8 weeks  Freq: Three times a week  Duration: 40min | CON | 5m sprint, 10m sprint, 30m sprint |
| Jovanovic | Croatia | 2011 | Soccer players | T:19(NA)  C:19(NA) | T:50/50/0  C:50/50/0 | Agility training  Length of Intervention: 8 weeks  Freq: Three times a week  Duration: 100min | CON | CMJ, 5m sprint, 10m sprint |
| Polman | UK | 2004 | Soccer players | T+C:21.2(3.1) | T:12/0/12  C:12/0/12 | Agility training  Length of Intervention: 12 weeks  Freq: Twice a week  Duration: 60min | CON | VO2max |
| Arslan | Turkey | 2020 | Soccer players | T+C:14.2(0.5) | T:10/10/0  C:10/10/0 | Endurance training  Length of Intervention: 5 weeks  Freq: Twice a week  Duration: 30min | CON | CMJ, SJ, 10m sprint, 30m sprint, VO2max |
| Cabo | Spain | 2020 | Soccer players | T:12.1(0.3)  C:12.5(0.5) | T:10/10/0  C:8/8/0 | Agility training  Length of Intervention: 6 weeks  Freq: Three times a week  Duration: 60min | CON | 10m sprint |
| Neag | Romania | 2025 | Soccer players | T:10.63(0.48)  C:10.89(0.31) | T:16/16/0  C:15/15/0 | Agility training  Length of Intervention: 18 months  Freq: Twice a week  Duration: 90min | CON | 10m sprint |
| Cabo | Spain | 2021 | Soccer players | T:12.6(0.7)  C:12.39(0.56) | T:10/10/0  C:10/10/0 | Agility training  Length of Intervention: 6 weeks  Freq: Twice a week  Duration: 70min | CON | CMJ, SJ, 5m sprint, 10m sprint |
| Alsharab | Tunisia | 2024 | Soccer players | T:17.32(1.02)  C:17.21(1.7) | T:19/19/0  C:19/19/0 | Strength training  Length of Intervention: 12 weeks  Freq: Three times a week  Duration: 60min | CON | CMJ, 30m sprint, T test, VO2max |
| Fernández | Spain | 2021 | Soccer players | T:14.5(0.51)  C:14.7(0.47) | T:20/20/0  C:20/20/0 | Agility training  Length of Intervention: 10 months  Freq: Three times a week  Duration: 60min | CON | CMJ, 30m sprint |
| Rella´n | Spain | 2024 | Soccer players | T+C:18.1(0.66) | T:18/18/0  C:12/12/0 | Speed training  Length of Intervention: 6 weeks  Freq: Twice a week  Duration: 90min | CON | 5m sprint, 10m sprint, 30m sprint, T test |
| Clemente | Portugal | 2022 | Soccer players | T:16.6(0.5)  C:16.3(0.5) | T:20/20/0  C:20/20/0 | Endurance training  Length of Intervention: 4 weeks  Freq: Three times a week  Duration: 80min | CON | CMJ, 5m sprint, 10m sprint |
| Lesinski | Germany | 2021 | Soccer players | T:15.3(0.5)  C:15.4(0.6) | T:17/0/17  C:16/0/16 | Endurance training  Length of Intervention: 38 weeks  Freq: Twice a week  Duration: 60min | CON | CMJ, 10m sprint, T test |
| Faude | Germany | 2014 | Soccer players | T+C:16.5(0.8) | T+C:19/19/0 | Endurance training  Length of Intervention: 4 weeks  Freq: Twice a week  Duration: 30min | CON | CMJ, 5m sprint, 10m sprint, 30m sprint |
| Nayıroğlu | Turkey | 2022 | Soccer players | T: 18.5(2.1)  C: 18.8(2.7) | T:11/0/11  C:12/0/12 | Endurance training  Length of Intervention: 8 weeks  Freq: Three times a week  Duration: 60min | CON | CMJ, 10m sprint, 30m sprint |
| Jastrzebski | Poland | 2014 | Soccer players | T:15.8(0.55)  C:15.8(0.63) | T:11/11/0  C:11/11/0 | Speed training  Length of Intervention: 8 weeks  Freq: Twice a week  Duration: 60min | CON | 5m sprint, 30m sprint, VO2max |
| Jlid | Tunisia | 2019 | Soccer players | T:11.8(0.4)  C:11.6(0.5) | T:14/14/0  C:14/14/0 | Speed training  Length of Intervention: 8 weeks  Freq: Twice a week  Duration: 60min | CON | CMJ, SJ, T test |
| Campo | Spain | 2011 | Soccer players | T:18.4(1.1)  C:18.2(0.9) | T:11/11/0  C:11/11/0 | Speed training  Length of Intervention: 10 weeks  Freq: Three times a week  Duration: 60min | CON | CMJ, SJ, 10m sprint |
| Michailidis | Spain | 2013 | Soccer players | T:10.9(0.7)  C:10.8(0.6) | T:24/24/0  C:21/21/0 | Speed training  Length of Intervention: 12 weeks  Freq: Twice a week  Duration: 60min | CON | CMJ, SJ, 10m sprint, 30m sprint, VO2max |
| Hammami | Tunisia | 2016 | Soccer players | T:15.7(0.2)  C:15.8(0.2) | T:15/15/0  C:13/13/0 | Speed training  Length of Intervention: 8 weeks  Freq: Twice a week  Duration: 60min | CON | 5m sprint, 10m sprint, 30m sprint |
| Milanović | Serbia | 2014 | Soccer players | T:18.5(0.4)  C:18.6(0.6) | T:66/66/0  C:66/66/0 | Agility training  Length of Intervention: 12 months  Freq: Four times a week  Duration: 90min | CON | 5m sprint, 10m sprint |
| Trecroci | Italy | 2016 | Soccer players | T:10.5(0.3)  C:10.7(0.21) | T:20/20/0  C:15/15/0 | Agility training  Length of Intervention: 12 months  Freq: Twice a week  Duration: 25min | CON | 5m sprint |
| Mackała | Poland | 2019 | Soccer players | T+C:17.63(0.48) | T:8/8/0  C:8/8/0 | Endurance training  Length of Intervention: 8 weeks  Freq: Once a week  Duration: 120min | CON | VO2max |
| Boraczyński | Poland | 2023 | Soccer players | T:25.6(3.98)  C:24.3(5.16) | T:13/13/0  C:12/12/0 | Endurance training  Length of Intervention: 7 weeks  Freq: Twice a week  Duration: 90min | CON | CMJ, 5m sprint, 30m sprint, VO2max |
| Ingebrigtsen | Norway | 2013 | Soccer players | T:16.9(0.4)  C:16.9(0.8) | T:8/8/0  C:8/8/0 | Endurance training  Length of Intervention: 6 weeks  Freq: Twice a week  Duration: 60min | CON | CMJ, SJ, 10m sprint |
| Haugen | Norway | 2014 | Soccer players | T+C:17(1) | T:13/6/7  C:9/5/4 | Endurance training  Length of Intervention: 9 weeks  Freq: Four times a week  Duration: 60min | CON | CMJ, VO2max |
| Sperlich | Germany | 2011 | Soccer players | T+C:13.5(0.4) | T:9/9/0  C:8/8/0 | Endurance training  Length of Intervention: 5 weeks  Freq: Three times a week  Duration: 30min | CON | CMJ, SJ, 30m sprint, VO2max |
| Arcos | Spain | 2015 | Soccer players | T:15.8(0.5)  C:15.1(0.7) | T:8/8/0  C:7/7/0 | Endurance training  Length of Intervention: 8 weeks  Freq: Three times a week  Duration: 60min | CON | CMJ |
| Impellizzeri | Italy | 2006 | Soccer players | T+C:17.2(0.8) | T:15/15/0  C:14/14/0 | Endurance training  Length of Intervention: 12 weeks  Freq: Twice a week  Duration: 60min | CON | VO2max |
| Bloomfield | UK | 2007 | Soccer players | T+C:20.5(3.1) | T:14/8/6  C:16/8/8 | Agility training  Length of Intervention: 6 weeks  Freq: Twice a week  Duration: 60min | CON | CMJ, 5m sprint, T test |

Note: CON: control group with routine exercise, T: experimental group, C: control group, CMJ: counter movement jump, SJ: squat jump, 1-RM squat: one rep max squat, 5m sprint: five meters sprint, 10m sprint: ten meters sprint, 30m sprint: thirty meters sprint, T test: t-test for agility, VO2max: maximal oxygen uptake, T+C: The ages of the experimental and control groups were not reported separately in the study, only the overall age was reported, NA: unavailable, Freq: frequency

**Table** **S3A.** League table on CMJ

| strength training | speed training | agility training | small-sided game training | regular training | endurance training |
| --- | --- | --- | --- | --- | --- |
| strength training | -1.73 (-3.71,0.25) | -1.78 (-4.82,1.26) | -2.07 (-5.33,1.19) | -2.55 (-3.92,-1.17) | -2.67 (-5.21,-0.14) |
| 1.73 (-0.25,3.71) | speed training | -0.05 (-3.20,3.10) | -0.34 (-3.95,3.26) | -0.82 (-2.42,0.78) | -0.94 (-3.92,2.03) |
| 1.78 (-1.26,4.82) | 0.05 (-3.10,3.20) | agility training | -0.29 (-4.54,3.96) | -0.77 (-3.48,1.95) | -0.89 (-4.62,2.84) |
| 2.07 (-1.19,5.33) | 0.34 (-3.26,3.95) | 0.29 (-3.96,4.54) | small-sided game training | -0.48 (-3.75,2.80) | -0.60 (-2.64,1.44) |
| 2.55 (1.17,3.92) | 0.82 (-0.78,2.42) | 0.77 (-1.95,3.48) | 0.48 (-2.80,3.75) | regular training | -0.12 (-2.69,2.44) |
| 2.67 (0.14,5.21) | 0.94 (-2.03,3.92) | 0.89 (-2.84,4.62) | 0.60 (-1.44,2.64) | 0.12 (-2.44,2.69) | endurance training |

| strength training | speed training | agility training | endurance training | regular training | small-sided game training |
| --- | --- | --- | --- | --- | --- |
| strength training | -0.74 (-2.59,1.10) | -1.49 (-6.65,3.67) | -2.53 (-5.51,0.45) | -2.81 (-4.32,-1.29) | -3.33 (-7.95,1.29) |
| 0.74 (-1.10,2.59) | speed training | -0.75 (-5.85,4.36) | -1.79 (-4.98,1.40) | -2.06 (-3.38,-0.75) | -2.59 (-7.35,2.17) |
| 1.49 (-3.67,6.65) | 0.75 (-4.36,5.85) | agility training | -1.04 (-6.79,4.70) | -1.32 (-6.25,3.61) | -1.84 (-8.59,4.90) |
| 2.53 (-0.45,5.51) | 1.79 (-1.40,4.98) | 1.04 (-4.70,6.79) | endurance training | -0.28 (-3.23,2.68) | -0.80 (-4.33,2.73) |
| 2.81 (1.29,4.32) | 2.06 (0.75,3.38) | 1.32 (-3.61,6.25) | 0.28 (-2.68,3.23) | regular training | -0.52 (-5.13,4.08) |
| 3.33 (-1.29,7.95) | 2.59 (-2.17,7.35) | 1.84 (-4.90,8.59) | 0.80 (-2.73,4.33) | 0.52 (-4.08,5.13) | small-sided game training |

**Table S3B.** League table on SJ

**Table S3C.** League table on 1-RM squat

| strength training | speed training | endurance training | regular training |
| --- | --- | --- | --- |
| strength training | -19.44 (-33.66,-5.21) | -20.75 (-30.70,-10.80) | -31.23 (-38.30,-24.17) |
| 19.44 (5.21,33.66) | speed training | -1.31 (-18.09,15.46) | -11.80 (-24.14,0.55) |
| 20.75 (10.80,30.70) | 1.31 (-15.46,18.09) | endurance training | -10.48 (-21.84,0.88) |
| 31.23 (24.17,38.30) | 11.80 (-0.55,24.14) | 10.48 (-0.88,21.84) | regular training |

**Table S3D.** League table on 5m-sprint

| strength training | agility training | speed training | regular training | small-sided game training | endurance training |
| --- | --- | --- | --- | --- | --- |
| strength training | 0.05 (0.01,0.10) | 0.05 (0.01,0.10) | 0.09 (0.06,0.12) | 0.13 (0.07,0.19) | 0.13 (0.08,0.18) |
| -0.05 (-0.10,-0.01) | agility training | 0.00 (-0.04,0.04) | 0.04 (0.01,0.07) | 0.08 (0.01,0.14) | 0.07 (0.01,0.13) |
| -0.05 (-0.10,-0.01) | -0.00 (-0.04,0.04) | speed training | 0.04 (0.00,0.07) | 0.08 (0.01,0.15) | 0.07 (0.01,0.14) |
| -0.09 (-0.12,-0.06) | -0.04 (-0.07,-0.01) | -0.04 (-0.07,-0.00) | regular training | 0.04 (-0.02,0.10) | 0.04 (-0.01,0.09) |
| -0.13 (-0.19,-0.07) | -0.08 (-0.14,-0.01) | -0.08 (-0.15,-0.01) | -0.04 (-0.10,0.02) | small-sided game training | -0.00 (-0.03,0.03) |
| -0.13 (-0.18,-0.08) | -0.07 (-0.13,-0.01) | -0.07 (-0.14,-0.01) | -0.04 (-0.09,0.01) | 0.00 (-0.03,0.03) | endurance training |

**Table S3E.** League table on 10m-sprint

| strength training | agility training | speed training | endurance training | regular training | small-sided game training |
| --- | --- | --- | --- | --- | --- |
| strength training | 0.01 (-0.06,0.07) | 0.02 (-0.03,0.08) | 0.09 (0.04,0.15) | 0.09 (0.05,0.13) | 0.10 (0.02,0.19) |
| -0.01 (-0.07,0.06) | agility training | 0.02 (-0.05,0.08) | 0.09 (0.01,0.16) | 0.09 (0.03,0.14) | 0.10 (-0.00,0.20) |
| -0.02 (-0.08,0.03) | -0.02 (-0.08,0.05) | speed training | 0.07 (0.00,0.14) | 0.07 (0.03,0.11) | 0.08 (-0.01,0.17) |
| -0.09 (-0.15,-0.04) | -0.09 (-0.16,-0.01) | -0.07 (-0.14,-0.00) | endurance training | 0.00 (-0.05,0.05) | 0.01 (-0.05,0.08) |
| -0.09 (-0.13,-0.05) | -0.09 (-0.14,-0.03) | -0.07 (-0.11,-0.03) | -0.00 (-0.05,0.05) | regular training | 0.01 (-0.07,0.10) |
| -0.10 (-0.19,-0.02) | -0.10 (-0.20,0.00) | -0.08 (-0.17,0.01) | -0.01 (-0.08,0.05) | -0.01 (-0.10,0.07) | small-sided game training |

**Table S3F.** League table on 30m-sprint

| strength training | speed training | agility training | regular training | endurance training | small-sided game training |
| --- | --- | --- | --- | --- | --- |
| strength training | 0.02 (-0.09,0.14) | 0.09 (-0.13,0.32) | 0.18 (0.09,0.27) | 0.30 (0.12,0.47) | 0.30 (0.08,0.53) |
| -0.02 (-0.14,0.09) | speed training | 0.07 (-0.15,0.30) | 0.15 (0.07,0.24) | 0.27 (0.08,0.46) | 0.28 (0.05,0.51) |
| -0.09 (-0.32,0.13) | -0.07 (-0.30,0.15) | agility training | 0.08 (-0.13,0.29) | 0.20 (-0.07,0.47) | 0.21 (-0.09,0.51) |
| -0.18 (-0.27,-0.09) | -0.15 (-0.24,-0.07) | -0.08 (-0.29,0.13) | regular training | 0.12 (-0.05,0.29) | 0.13 (-0.09,0.34) |
| -0.30 (-0.47,-0.12) | -0.27 (-0.46,-0.08) | -0.20 (-0.47,0.07) | -0.12 (-0.29,0.05) | endurance training | 0.01 (-0.13,0.14) |
| -0.30 (-0.53,-0.08) | -0.28 (-0.51,-0.05) | -0.21 (-0.51,0.09) | -0.13 (-0.34,0.09) | -0.01 (-0.14,0.13) | small-sided game training |

**Table S3G.** League table on T-test

| strength training | speed training | agility training | endurance training | regular training |
| --- | --- | --- | --- | --- |
| strength training | 0.07 (-1.09,1.22) | 0.04 (-1.49,1.56) | 0.37 (-0.55,1.28) | 0.63 (-0.16,1.43) |
| -0.07 (-1.22,1.09) | speed training | -0.03 (-1.33,1.28) | 0.30 (-1.03,1.63) | 0.57 (-0.27,1.41) |
| -0.04 (-1.56,1.49) | 0.03 (-1.28,1.33) | agility training | 0.33 (-1.33,1.98) | 0.60 (-0.71,1.90) |
| -0.37 (-1.28,0.55) | -0.30 (-1.63,1.03) | -0.33 (-1.98,1.33) | endurance training | 0.27 (-0.76,1.29) |
| -0.63 (-1.43,0.16) | -0.57 (-1.41,0.27) | -0.60 (-1.90,0.71) | -0.27 (-1.29,0.76) | regular training |

**Table S3H.** League table on VO2max

| endurance training | small-sided game training | strength training | speed training | regular training | agility training |
| --- | --- | --- | --- | --- | --- |
| endurance training | -0.04 (-0.78,0.70) | -0.60 (-2.01,0.81) | -2.18 (-4.97,0.61) | -1.94 (-3.09,-0.79) | -2.34 (-5.02,0.34) |
| 0.04 (-0.70,0.78) | small-sided game training | -0.57 (-2.16,1.03) | -2.14 (-5.03,0.74) | -1.90 (-3.27,-0.54) | -2.30 (-5.08,0.47) |
| 0.60 (-0.81,2.01) | 0.57 (-1.03,2.16) | strength training | -1.58 (-4.24,1.09) | -1.34 (-2.16,-0.52) | -1.74 (-4.29,0.81) |
| 2.18 (-0.61,4.97) | 2.14 (-0.74,5.03) | 1.58 (-1.09,4.24) | speed training | 0.24 (-2.30,2.78) | -0.16 (-3.67,3.35) |
| 1.94 (0.79,3.09) | 1.90 (0.54,3.27) | 1.34 (0.52,2.16) | -0.24 (-2.78,2.30) | regular training | -0.40 (-2.82,2.02) |
| 2.34 (-0.34,5.02) | 2.30 (-0.47,5.08) | 1.74 (-0.81,4.29) | 0.16 (-3.35,3.67) | 0.40 (-2.02,2.82) | agility training |

**Table S3.** League table for outcomes: A: League table on CMJ; B: League table on SJ; C: League table on 1-RM squat; D: League table on 5m-sprint; E: League table on 10m-sprint; F: League table on 30m-sprint; G: League table on T-test; H: League table on VO2max.

**Table S4A.** Node-splitting result on CMJ.

**Side Direct Indirect Difference tau**

**Coef. Std. Err. Coef. Std. Err. Coef. Std. Err. P>|z|**

A C * -.7657227 1.384291 .0326857 57.74504 -.7984084 57.76161 0.989 2.048136

B C .7472484 1.844922 -.5061081 1.880023 1.253356 2.632865 0.634 2.082669

B D * .6004354 1.040722 2.380022 258.1831 -1.779587 258.1837 0.995 2.048117

B F 2.141425 1.729611 3.394247 1.987854 -1.252822 2.632858 0.634 2.082671

C E .8349425 .8932383 .7084004 2.359452 .1265421 2.522858 0.960 2.107296

C F 2.646291 .7808762 2.064777 1.744037 .5815145 1.911324 0.761 2.097715

E F 1.830002 2.237719 1.703276 1.16539 .1267259 2.522999 0.960 2.107302

**Table S4B.** Node-splitting result on SJ.

**Side Direct Indirect Difference tau**

**Coef. Std. Err. Coef. Std. Err. Coef. Std. Err. P>|z|**

A C * -1.320002 2.517219 -.2308679 74.53146 -1.089134 74.57395 0.988 1.690264

B C -1.120604 2.021917 .8243626 2.300708 -1.944966 3.067041 0.526 1.709058

B D * -.8000031 1.802444 1.286756 632.9132 -2.086759 632.9152 0.997 1.690208

B F 3.499988 2.154657 1.554764 2.182704 1.945224 3.067046 0.526 1.70906

C E 1.820252 .7112969 4.011732 2.006107 -2.19148 2.127509 0.303 1.684227

C F 3.018284 .8943381 2.103272 1.681089 .9150116 1.901312 0.630 1.753375

E F -.869995 1.824245 1.321498 1.094905 -2.191493 2.127601 0.303 1.684234

**Table S4C.** Node-splitting result on 1-RM squat.

**Side Direct Indirect Difference tau**

**Coef. Std. Err. Coef. Std. Err. Coef. Std. Err. P>|z|**

A B -12.79999 11.32471 -9.658627 6.74639 -3.141361 13.18191 0.812 6.03e-09

A D 21.3 5.611001 18.28283 11.89221 3.01717 13.14945 0.819 3.84e-08

B C * 11.79589 6.297012 32.77287 447.4267 -20.97698 447.3824 0.963 3.59e-10

B D 30.98191 3.762057 34.04563 12.62186 -3.063723 13.17162 0.816 4.60e-08

**Table S4D.** Node-splitting result on 5m-sprint.

**Side Direct Indirect Difference tau**

**Coef. Std. Err. Coef. Std. Err. Coef. Std. Err. P>|z|**

A C * -.0366609 .0144964 .0104559 93.71555 -.0471168 93.71557 1.000 .0222951

B C -.01 .0351653 .0896408 .0358723 -.0996408 .0502336 0.047 .0185926

B D * -.0008643 .0164134 -73.68725 1.899076 73.68639 1.899111 0.000 .0222985

B F .17 .0324655 .0703592 .0383324 .0996408 .0502333 0.047 .0185924

C E * .0363928 .0168517 -23.77965 3.564689 23.81605 3.564677 0.000 .0222976

C F .0803592 .0152579 .18 .0478602 -.0996408 .0502335 0.047 .0185925

**Table S4E.** Node-splitting result on 10m-sprint.

**Side Direct Indirect Difference tau**

**Coef. Std. Err. Coef. Std. Err. Coef. Std. Err. P>|z|**

A C * -.0850213 .0271615 -.0014975 76.31456 -.0835238 76.31458 0.999 .0511519

B C -.0355119 .0343687 .0610234 .0454766 -.0965353 .0570312 0.091 .0498063

B D * .0059039 .0290485 .4153493 251.4018 -.4094454 251.4018 0.999 .051152

B F .1395553 .0403205 .0430201 .0402872 .0965352 .0570323 0.091 .0498062

C E * .0691268 .0194328 .5920937 121.6786 -.522967 121.6786 0.997 .0511525

C F .0785321 .021019 .1750664 .05301 -.0965343 .0570315 0.091 .0498059

**Table S4F.** Node-splitting result on 30m-sprint.

**Side Direct Indirect Difference tau**

**Coef. Std. Err. Coef. Std. Err. Coef. Std. Err. P>|z|**

A C * -.0825501 .1068017 .0259764 61.85355 -.1085265 61.85363 0.999 .1177839

B C .0553461 .1054478 .2450247 .1501122 -.1896787 .1834718 0.301 .1168593

B D * -.0088767 .0688836 .2797119 409.4741 -.2885886 409.4741 0.999 .1178006

B F .4099998 .1426163 .2203208 .1154231 .1896791 .1834718 0.301 .1168593

C E .1678643 .0457999 .0232113 .1456648 .144653 .1526773 0.343 .1176671

C F .1474315 .048069 .3264318 .1086388 -.1790003 .1187883 0.132 .1119462

E F .1400003 .1375459 -.0046528 .0662682 .1446532 .1526773 0.343 .1176671

**Table S4G.** Node-splitting result on T-test.

**Side Direct Indirect Difference tau**

**Coef. Std. Err. Coef. Std. Err. Coef. Std. Err. P>|z|**

A C -.0299997 .8891343 -1.316915 1.004815 1.286915 1.34172 0.337 .7599982

A D -.5999994 .894881 .6870252 .9997131 -1.287025 1.34173 0.337 .7599985

B C .4099997 .7412163 -.8391524 .6794676 1.249152 1.005524 0.214 .7224276

B E .0233515 .5251642 1.2725 .8574577 -1.249149 1.005524 0.214 .7224277

C D .7170259 .4570867 -.569997 1.261483 1.287023 1.341741 0.337 .7600003

C E .862503 .4310839 -.3866164 .9083959 1.249119 1.005517 0.214 .722427

**Table S4H.** Node-splitting result on VO2max.

**Side Direct Indirect Difference tau**

**Coef. Std. Err. Coef. Std. Err. Coef. Std. Err. P>|z|**

A C * .4000015 1.234052 -1.234546 95.32836 1.634547 95.33635 0.986 4.60e-08

B C * -1.940042 .585748 1.300001 175.5946 -3.240043 175.5964 0.985 1.66e-07

B D * -.0363958 .37721 -4.717312 441.7458 4.680916 441.7456 0.992 1.39e-07

C E * -.2392419 1.295057 -1.038722 447.8752 .7994801 447.8733 0.999 1.09e-07

C F * 1.33851 .4171938 .5390146 447.9738 .7994958 447.9737 0.999 4.86e-09

Table S5. Search strategy on Embase.

| #1 | football:ab,ti OR soccer:ab,ti OR 'american football':ab,ti OR 'football, american':ab,ti OR 'football european':ab,ti OR 'european, football':ab,ti OR 'europeans, football':ab,ti OR 'european football':ab,ti OR 'football, european':ab,ti |
| --- | --- |
| #2 | 'physical education':ab,ti AND training:ab,ti OR 'physical conditioning, human':ab,ti OR exercise:ab,ti OR 'physical education, training':ab,ti OR 'physical education':ab,ti OR 'education, physical':ab,ti OR 'conditioning, human physical':ab,ti OR 'human physical conditioning':ab,ti OR 'physical training, human':ab,ti OR 'human physical training':ab,ti OR 'training, human physical':ab,ti OR 'physical exercise':ab,ti OR 'isometric exercises':ab,ti OR 'exercise training':ab,ti OR 'physical activities':ab,ti OR training:ab,ti OR intervention:ab,ti OR strength:ab,ti OR power:ab,ti OR explosive:ab,ti OR resistance:ab,ti OR 'velocity-based resistance':ab,ti OR ballistic:ab,ti OR plyometric:ab,ti OR 'offset load':ab,ti OR trx:ab,ti OR speed:ab,ti OR jump:ab,ti OR 'linear speed':ab,ti OR 'multidirectional speed':ab,ti OR 'acceleration speed':ab,ti OR 'reaction speed':ab,ti OR 'running economy':ab,ti OR endurance:ab,ti OR 'high-intensity interval training':ab,ti OR 'moderate-intensity continuous training':ab,ti OR 'long slow distance':ab,ti OR coordination:ab,ti OR flexibility:ab,ti OR 'static stretching':ab,ti OR 'dynamic stretching':ab,ti OR 'proprioceptive neuromuscular facilitation':ab,ti OR agility:ab,ti OR 'multimodal agility-based exercise training':ab,ti OR saq:ab,ti |
| #3 | #1 AND #2 |
| #4 | 'randomized controlled':ab,ti OR random:ab,ti OR rct:ab,ti OR 'clinical trial':ab,ti |
| #5 | #3 AND #4 |

Table S6. Search strategy on Web of Science.

| #1 | ((((((((TI=(Football)) OR TI=(Soccer)) OR TI=(American Football)) OR TI=(Football, American)) OR TI=(Football European)) OR TI=(European, Football)) OR TI=(Europeans, Football)) OR TI=(European Football)) OR TI=(Football, European) |
| --- | --- |
| #2 | ((((((((((((((((((((((((((((((((((((((((((((TI=(Physical Education and Training)) OR TI=(Physical Conditioning, Human)) OR TI=(Exercise)) OR TI=(Physical Education, Training)) OR TI=(Physical Education)) OR TI=(Education, Physical)) OR TI=(Conditioning, Human Physical)) OR TI=(Human Physical Conditioning)) OR TI=(Physical Training, Human)) OR TI=(Human Physical Training)) OR TI=(Training, Human Physical)) OR TI=(Physical Exercise)) OR TI=(Isometric Exercises)) OR TI=(Exercise Training)) OR TI=(Physical Activities)) OR TI=(Training)) OR TI=(Intervention)) OR TI=(strength)) OR TI=(power)) OR TI=(explosive)) OR TI=(resistance)) OR TI=(velocity-based resistance)) OR TI=(ballistic )) OR TI=(plyometric)) OR TI=(offset load )) OR TI=(TRX )) OR TI=(Speed)) OR TI=(Jump)) OR TI=(linear speed )) OR TI=(multidirectional speed )) OR TI=(acceleration-speed )) OR TI=(reaction speed)) OR TI=(running economy)) OR TI=(endurance)) OR TI=(high-intensity interval training)) OR TI=(moderate-intensity continuous training)) OR TI=(long slow distance)) OR TI=(coordination)) OR TI=(flexibility)) OR TI=(static stretching )) OR TI=(dynamic stretching)) OR TI=(proprioceptive neuromuscular facilitation)) OR TI=(agility)) OR TI=(multimodal agility-based exercise training)) OR TI=(SAQ) |
| #3 | #1 AND #2 |
| #4 | (((TI=(randomized controlled)) OR TI=(random)) OR TI=(RCT)) OR TI=(clinical trial) |
| #5 | #3 AND #4 |
